# Anaplastic Lymphoma Kinase signaling stabilizes SLC3A2 expression via MARCH11 to promote neuroblastoma cell growth

**DOI:** 10.1038/s41418-024-01319-0

**Published:** 2024-06-10

**Authors:** Wei-Yun Lai, Tzu-Po Chuang, Marcus Borenäs, Dan E. Lind, Bengt Hallberg, Ruth H. Palmer

**Affiliations:** Department of Medical Biochemistry and Cell Biology, Institute of Biomedicine, Sahlgrenska Academy, University of Gothenburg, SE-40530 Gothenburg, Sweden

**Keywords:** Paediatric cancer, Translational research

## Abstract

Solute Carrier Family 3, Member 2 (SLC3A2 or 4F2hc) is a multifunctional glycoprotein that mediates integrin-dependent signaling, acts as a trafficking chaperone for amino acid transporters, and is involved in polyamine transportation. We identified SLC3A2 as a potential Anaplastic Lymphoma Kinase (ALK) interacting partner in a BioID-proximity labeling screen in neuroblastoma (NB) cells. In this work we show that endogenous SLC3A2 and ALK interact in NB cells and that this SLC3A2:ALK interaction was abrogated upon treatment with the ALK inhibitor lorlatinib. We show here that loss of ALK activity leads to decreased SLC3A2 expression and reduced SLC3A2 protein stability in a panel of NB cell lines, while stimulation of ALK with ALKAL2 ligand resulted in increased SLC3A2 protein levels. We further identified MARCH11, an E3 ligase, as a regulator of SLC3A2 ubiquitination downstream of ALK. Further, knockdown of SLC3A2 resulted in inhibition of NB cell growth. To investigate the therapeutic potential of SLC3A2 targeting, we performed monotreatment of NB cells with AMXT-1501 (a polyamine transport inhibitor), which showed only moderate effects in NB cells. In contrast, a combination lorlatinib/AMXT-1501 treatment resulted in synergistic inhibition of cell growth in ALK-driven NB cell lines. Taken together, our results identify a novel role for the ALK receptor tyrosine kinase (RTK), working in concert with the MARCH11 E3 ligase, in regulating SLC3A2 protein stability and function in NB cells. The synergistic effect of combined ALK and polyamine transport inhibition shows that ALK/MARCH11/SLC3A2 regulation of amino acid transport is important for oncogenic growth and survival in NB cells.

## Introduction

SLC3A2 is a cell surface, transmembrane multifunctional glycoprotein that mediates integrin-dependent signaling, acts as a trafficking chaperone for amino acid transporters, and is involved in polyamine transport [1–5]. The SLC3A2 protein forms a heterodimeric complex with the SLC7 family transporters (SLC7A5, SLC7A6, SLC7A7, SLC7A8, SLC7A10, SLC7A11) and the glucose transporter 1 (SLC2A1) [1, 2, 6, 7]. The SLC3A2 heavy chain is required for the stability of the transporter at the plasma membrane, while the light chain facilitates amino acid transport across the membrane [1, 2, 7]. Notably, the SLC7-SLC3A2 transporter complex has been reported to be upregulated in cancer cells [8–11], where it plays a role in cancer growth and progression, supporting various processes regulated by the MYC family of oncoproteins [4, 7, 12, 13]. Additionally, elevated levels of polyamines in cancer cells contribute to poor prognosis and render the polyamine pathway an attractive therapeutic target [14].

NB is a pediatric neural crest-derived solid tumor with a high cancer-related mortality [15], and is heterogeneous, harboring diagnostically important genetic characteristics [16–18]. Few recurrent oncogenic mutations have been observed in NB, with one exception being activating point mutations in the Anaplastic Lymphoma Kinase (ALK) receptor tyrosine kinase (RTK) encoded by *ALK* [19–24]. Additional mutations have also been observed in genes such as *ATRX* and *PTPN11* [16, 18]. Rather, NB is characterized by genomic aberrations such as *MYCN* amplification, deletions of parts of chromosome 11q, 1p, gain of 2p and 17q and structural variants, e.g., in *TERT*, *DLG2,* and *PTPRD* [18, 25–28].

Relapsed neuroblastoma exhibiting both *ALK* mutation and *MYCN* amplification is regarded as a clinically challenging high-risk patient group with low survival rates [16, 29]. To identify ALK driven oncogenic factors that can be explored therapeutically it is important to understand underlying molecular signaling mechanisms and ALK interacting molecules. Earlier work, including proteomic and phosphoproteomic approaches has identified numerous ALK downstream signal transduction components, such as ETVs, IRS1/2, SHC, c-Cbl, PLCgamma, ATR, CHK1, SHP2, FRS2 and growth and cancer promoting pathways like RAS/MAPK, PI3K/AKT/mTOR/ERK5 [24, 30–40]. These studies have been complemented by recent proximity labeling BioID experiments to define an ALK interactome in NB cell lines [41], that confirmed many previously identified ALK signal transduction components, such as the IRS2, SHC, GAB1, FRS2, CRK and CRKL adapter proteins, the pseudokinase PEAK1 and the protein tyrosine phosphatase SHP2. In addition, this analysis revealed several intriguing proteins that have not previously been connected to ALK [41]. One of these was the Solute Carrier Family 3 (activators of dibasic and neutral amino acid transport), Member 2 (SLC3A2, 4F2hc, or CD98hc, accession number P08195) [41].

Given our BioID identification of SLC3A2 as a transmembrane glycoprotein in close proximity to ALK and previous findings showing SLC3A2 is involved in polyamine uptake in NB  [4], we explored the interaction between ALK and SLC3A2 in NB cells. Our results show that ALK interacts with and regulates SLC3A2 protein stability in a manner dependent on ALK activity in NB cells. Further, we show that the SLC3A2/ALK complex also includes the MARCH11 E3 ligase. Inhibition of ALK activity leads to the activation of MARCH11 and the subsequent ubiquitination and degradation of SLC3A2, regulating amino acid transporter stability and function downstream of ALK. Moreover, combined ALK and polyamine transport inhibition synergistically inhibits NB cell growth suggesting that the ALK/MARCH11/SLC3A2 regulatory module is important for NB cell survival and identifying a novel relationship between ALK and the regulation of amino acid transporters.

## Results

### The SLC3A2 and ALK interaction promotes SLC3A2 protein stability in NB cells

**Fig. 1:**
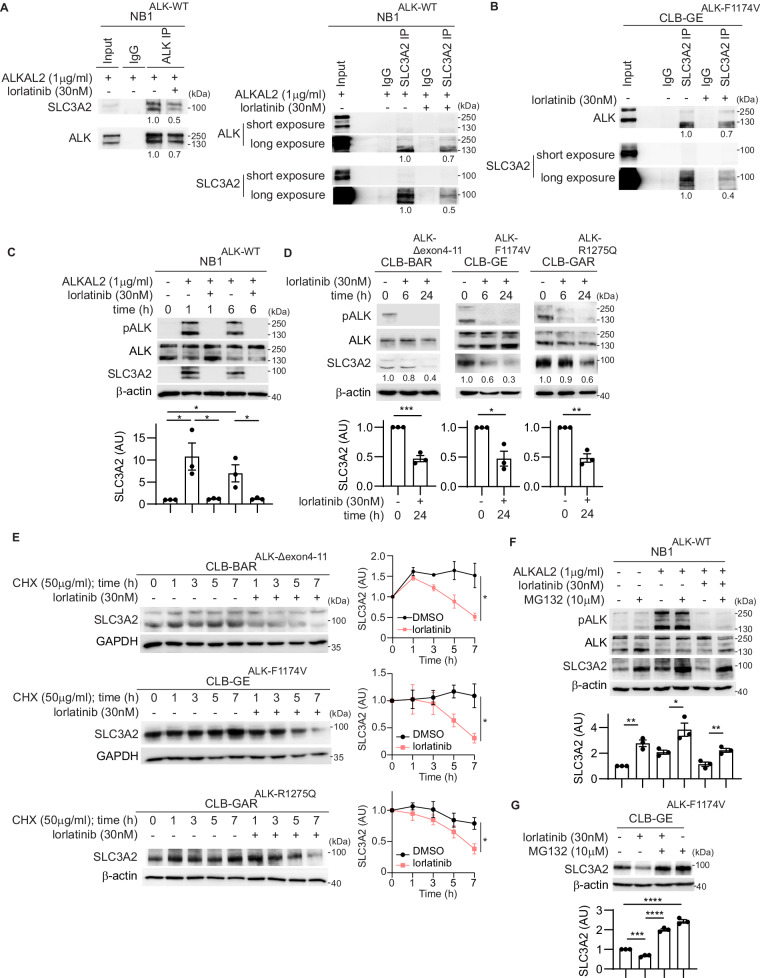
ALK signaling affects SLC3A2 protein stability. **A** NB1 cells were treated with ALKAL2 (1 µg/ml) and lorlatinib (0 or 30 nM) for 24 h. Lysates were subjected to immunoprecipitation with anti-ALK or anti-SLC3A2 antibodies followed by immunoblotting for ALK and SLC3A2. Anti-rabbit IgG was employed as negative control. **B** CLB-GE cells were treated with lorlatinib (0 or 30 nM) for 24 h. Lysates were subjected to immunoprecipitation with anti-SLC3A2 antibodies followed by immunoblotting for ALK and SLC3A2. Anti-rabbit IgG was employed as negative control. **C** Time-course of NB1 cells treated with ALKAL2 (1 µg/ml) and lorlatinib (0 or 30 nM) for 0, 1 and 6 h. Lysates were immunoblotted for ALK, pY1278-ALK and SLC3A2. β-actin was employed as loading control. **D** Time-course of CLB-BAR, CLB-GE and CLB-GAR cells treated with lorlatinib (30 nM). Lysates were immunoblotted for ALK, pY1278-ALK and SLC3A2. β-actin was employed as loading control. **E** CLB-BAR, CLB-GE, and CLB-GAR cells were treated with CHX (50 µg/ml) and lorlatinib (0 or 30 nM) for the times indicated. SLC3A2 and β-actin protein levels were detected by immunoblotting and quantified by ImageJ software (n = 3, mean ± s.e.m.). NB1 cells were treated with ALKAL2 (1 µg/ml), lorlatinib (0 or 30 nM), and MG132 (0 or 10 µM) for 24 h (**F**). CLB-GE cells were treated with lorlatinib (0 or 30 nM) and MG132 (0 or 10 µM) for 24 h (**G**). Lysates were immunoblotted for ALK, pY1278-ALK and SLC3A2 as indicated. β-actin was employed as loading control. Immunoblot experiments were performed in biologically independent triplicates. Quantification was performed with ImageJ and analyzed by *Student t*-test (unpaired, two-tailed). *p*-values are indicated (****P < 0.0001, ***0.0001 < *P* < 0.001, **0.001 < *P* < 0.01, *0.01 < *P* < 0.05, ns *P* ≥ 0.05); exact *P*-values are shown in Supplementary Fig. 1.

SLC3A2 was previously identified as a potential interacting partner with ALK in a BioID-based in vivo proximity labeling study in SK-N-AS.ALK-BirA* NB cells [41]. To further investigate the interaction between SLC3A2 and ALK, anti-ALK and anti-SLC3A2 co-immunoprecipitation was performed in NB1 (*ALK-WT*) and CLB-GE (*ALK-F1174V*) NB cells. A robust ALK:SLC3A2 interaction was observed in both cell lines (Fig. 1A, B, Sup. Fig. 1A, B). Further, we could also verify that SLC3A2 is present in complex with SLC7A5 and SLC7A11 as previously reported (Sup. Fig. 1C) [42–44]. Having validated the ALK:SLC3A2 interaction in two independent NB cell lines, we investigated the effect of ALK signaling on SLC3A2 protein levels. Activation of ALK with ALKAL2 ligand resulted in a robust increase in SLC3A2 protein levels that was observed at 1 h post-ALKAL2 stimulation. In keeping with an important role of ALK activity, this effect was blocked by the addition of lorlatinib (Fig. 1C, Sup. Fig. 1D). Treatment of three additional NB cell lines, CLB-BAR (*ALK-Δexon4-11*), CLB-GE (*ALK-F1174V*) and CLB-GAR (*ALK-R1275Q*), confirmed that ALK signaling is required for SLC3A2 protein stabilization in a range of NB cell lines (Fig. 1D, Sup. Fig. 1E). To further investigate the effect of ALK signaling on SLC3A2 protein stability, cycloheximide (CHX) chase assays were performed in the presence or absence of lorlatinib in NB cell lines harboring ALK activating mutations. SLC3A2 protein levels were stable in untreated NB cell lines, however, addition of lorlatinib resulted in a significant decrease in the half-life of SLC3A2 to 7.6 ± 0.96 h in CLB-BAR, 5.5 ± 0.83 h in CLB-GE, and 6.2 ± 0.83 h in CLB-GAR (Fig. 1E, Sup. Fig. 1F). Treatment of NB1 and CLB-GE cell lines with the proteasome inhibitor MG132 for 24 h, with or without lorlatinib treatment, resulted in a significant increase in SLC3A2 protein levels. This effect was observed in either absence or presence of ALKAL2 stimulation in NB1 cells. These findings indicate that SLC3A2 degradation occurs through the proteasome pathway (Fig. 1F, G, Sup. Fig. 1G, H).

### ALK signaling regulates SLC3A2 protein stability through ubiquitination

**Fig. 2:**
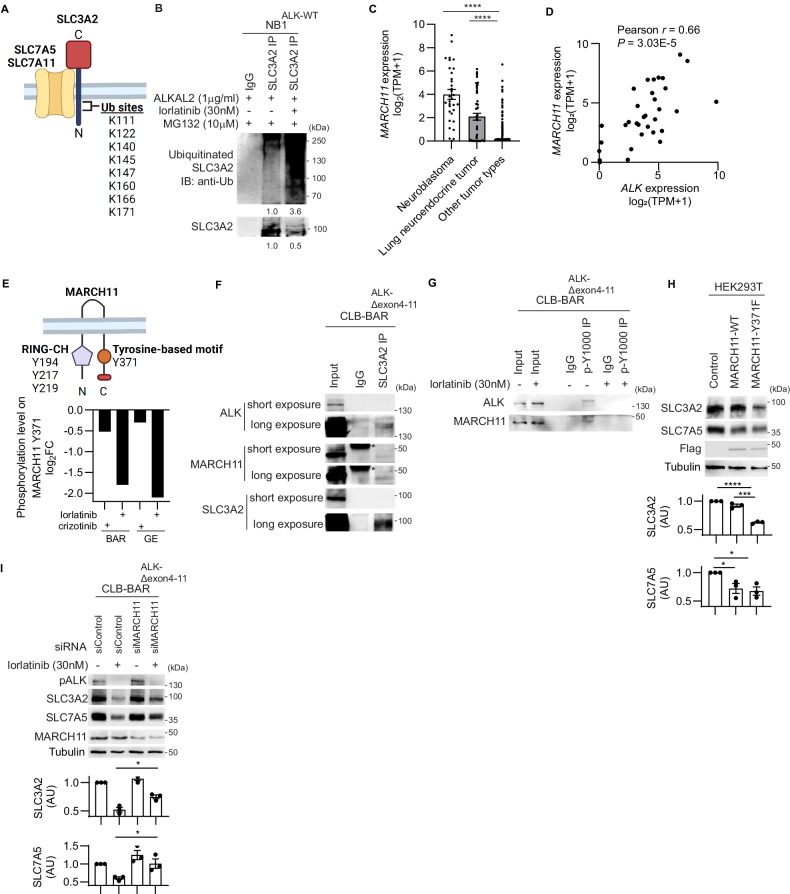
ALK signaling regulates SLC3A2 ubiquitination via MARCH11. **A** Schematic indicating potential lysine (K) ubiquitination sites in the SLC3A2 N-terminal tail (created with BioRender.com). **B** NB1 cells were treated with ALKAL2 (1 µg/ml), lorlatinib (0 or 30 nM) and MG132 (10 µM) for 24 h. Lysates were subjected to immunoprecipitation with anti-SLC3A2 antibodies and immunoprecipitated complexes were immunoblotted for SLC3A2 and ubiquitin (Ub). Anti-rabbit IgG was employed as control for immunoprecipitation. **C** *MARCH11* RNA expression levels in NB (n = 33), lung neuroendocrine tumor (n = 53) and other tumor types (n = 1364) derived from DepMap (https://depmap.org/portal/). **D** Correlation of *ALK* and *MARCH11* RNA expression levels in NB (n = 33) derived from DepMap (https://depmap.org/portal/). Pearson correlation test. **E** Schematic indicating protein domains and putative tyrosine phosphorylation sites in MARCH11 (created with BioRender.com). MARCH11 is composed of an N-terminal RING C4HC3-type domain (RING-CH, purple), two transmembrane domains, one tyrosine-based motif (YVLL, orange), and one C-terminal PDZ-binding domain (red). Phosphorylation of MARCH11 Y371 in CLB-BAR and CLB-GE cells in response to ALK inhibitor (crizotinib or lorlatinib) treatment for 1 h detected by LC-MS/MS analysis [38]. CLB-BAR cells were treated with lorlatinib (0 or 30 nM) for 6 h and lysates subjected to immunoprecipitation with anti-SLC3A2 or p-Y1000 antibodies. Immunoblotting was performed to detect ALK, MARCH11 and SLC3A2 (**F**), and phosphorylation of ALK and MARCH11 (**G**). IgG heavy chains are indicated with asterisk (*). **H** HEK293T cells were lysed 72 h after transfection with either vector control, MARCH11-WT or MARCH11-Y371F. Lysates were immunoblotted for SLC3A2, SLC7A5, and Flag. Tubulin was employed as loading control. **I** CLB-BAR cells were treated with either control siRNA or MARCH11-targeting pool siRNAs for 24 h, followed by lorlatinib (30 nM) treatment for an additional 24 h. Lysates were immunoblotted for pY1278-ALK, SLC3A2, SLC7A5, and MARCH11. Tubulin was employed as loading control. Immunoblot experiments were performed in biologically independent triplicates. Quantification was performed with ImageJ and analyzed by Student *t* test (unpaired, two-tailed). *p*-values are indicated (****P < 0.0001, ***0.0001 < *P* < 0.001, **0.001 < *P* < 0.01, *0.01 < *P* < 0.05, ns *P* ≥ 0.05); exact *P*-values are shown in Supplementary Fig. 2 and Supplementary Table 1.

Previous studies have shown that SLC3A2 protein stability is regulated through ubiquitination-mediated protein degradation [11]. SLC3A2 contains 8 lysines within its intracellular domain that are putative ubiquitination sites (Fig. 2A). Further, it has been reported that expression of either catalytically inactive Membrane-Associated Ring-CH type finger (MARCH)8 to prevent SLC3A2 ubiquitylation, or ubiquitylation-resistant SLC3A2, prevents MARCH8-induced SLC3A2 downregulation in human cells [45], prompting us to investigate SLC3A2 ubiquitination downstream of ALK. Immunoprecipitation of SLC3A2 from NB1 cells treated with lorlatinib for 24 h in the presence of MG132 resulted in an increase of ubiquitinated SLC3A2 in NB1 cells (Fig. 2B, Sup. Fig. 2A). Treatment with lorlatinib, was accompanied by a decrease in total SLC3A2 protein levels, in keeping with our earlier observations (Fig. 1C, D). There are 11 proteins in the MARCH protein family [46], that are differentially expressed at the mRNA level in NB cell lines (n = 33) on Depmap portal (https://depmap.org/portal/) (Sup. Fig. 2B). We noted that *MARCH11* expression is specifically enriched in NB and neuroendocrine tumors of the lung (https://depmap.org/portal/) (Fig. 2C) and *MARCH11* expression correlated significantly with *ALK* expression in NB cell lines (https://depmap.org/portal/)(Pearson *r* = 0.66, Fig. 2D). From our previous phosphoproteome analysis [38], we observed that 1 h treatment with either crizotinib or lorlatinib, first and third generation ALK inhibitors respectively, led to dephosphorylation of MARCH11 on Y371 in both CLB-BAR and CLB-GE cells, supporting a potential role for MARCH11 in the ubiquitination of SLC3A2 (Fig. 2E). To test this hypothesis, we employed anti-SLC3A2 immunoprecipitation and identified both ALK and MARCH11 in SLC3A2 complexes, suggesting that ALK:SLC3A2:MARCH11 form a complex in NB cells (Fig. 2F, Sup. Fig. 2C). Moreover, it has previously been shown that the E3 ligase activity of MARCH is negatively regulated by phosphorylation in colorectal carcinoma cells [46, 47]. Employing anti-p-Y1000 immunoprecipitation, we were able to show that MARCH11 was tyrosine phosphorylated in ALK-mutant NB cells, and that this was decreased upon treatment with the ALK inhibitor, lorlatinib (Fig. 2G, Sup. Fig. 2D). To investigate potential MARCH11 regulation by tyrosine phosphorylation downstream of ALK we generated MARCH11 wild type and Y371F mutant versions. HEK293T cells transfected with MARCH11-Y371F exhibited decreased levels of SLC3A2 protein compared with MARCH11-WT and vector controls (Fig. 2H, Sup. Fig. 2E).

To investigate the importance of MARCH11 for SLC3A2/SLC7A5 stability, CLB-BAR cells were treated with MARCH11 siRNA and SLC3A2 and SLC7A5 protein levels examined. A significant increase in SLC3A2 and SLC7A5 protein levels were observed on treatment with lorlatinib in MARCH11 knockdown CLB-BAR cells compared to controls (Fig. 2I, Sup. Figure 2F), supporting a role of MARCH11 as a negative regulator of SLC3A2/SLC7A5 protein stability. Taken together, these data suggest that ALK signaling maintains MARCH11 in an inactive state. Consequently, treatment with ALK inhibitors results in MARCH11 dephosphorylation and subsequent ubiquitination of SLC3A2 that promotes its degradation.

### Knockdown of SLC3A2 inhibits proliferation

**Fig. 3:**
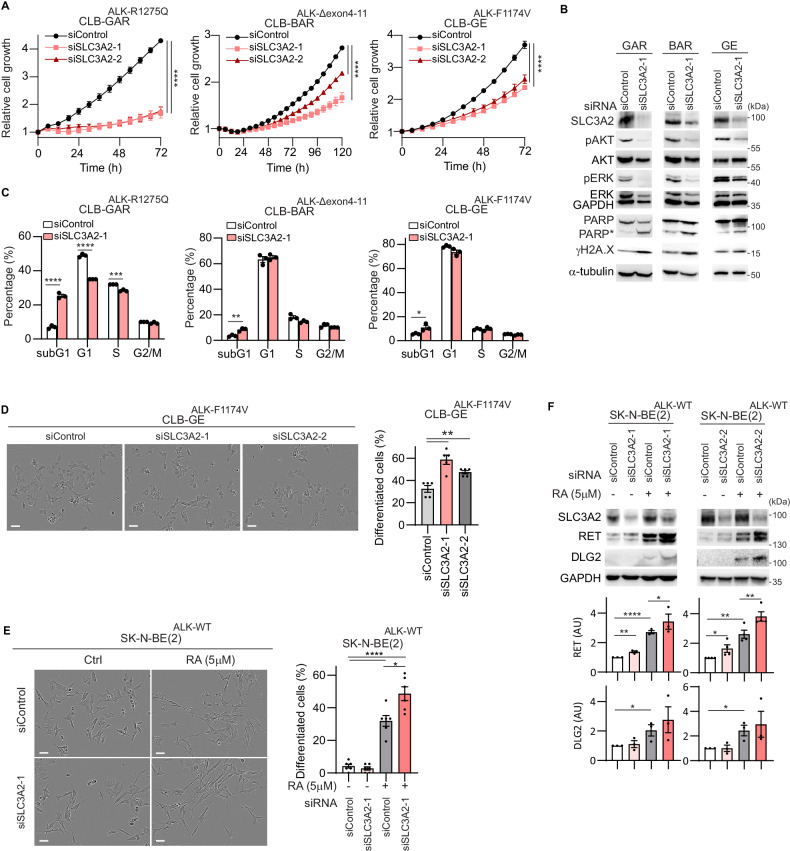
Knockdown of SLC3A2 suppresses NB cell growth and increases retinoic acid-induced neuronal differentiation. **A**–**D** CLB-BAR, CLB-GE, and CLB-GAR cells were treated with either control siRNA or SLC3A2-targeting siRNAs. **A** Cell growth was monitored by Incucyte every 6 h for 72-120 h (n = 3, mean ± s.e.m.). **B** Lysates collected at 72 h after siRNA transfection were immunoblotted with anti-SLC3A2, pAKT(S473), AKT, pERK (T202/Y204), ERK, PARP (asterisk (*) marked cleaved PARP), γH2A.X, α-tubulin and GAPDH. GAPDH was employed as immunoblotting loading control. Quantification of cleaved PARP (PARP*) and γH2A.X were performed with ImageJ (n = 3), shown in Supplementary Fig. 3. **C** Cell cycle analysis performed by NucleoCounter NC-3000 after 72 h (n = 3, mean ± s.e.m.). **D** Representative images showing differentiation morphology of CLB-GE cells 72 h post siRNA transfection. Scale bar = 50 µm. Bar graph indicates percentage of differentiated cells following siRNA treatment (n = 5, mean ± s.e.m). **E**, **F** SK-N-BE(2) cells treated with either SLC3A2-targeting or control siRNAs for 24 h, followed by retinoic acid (RA, 5 µM) for 48 h. **E** Representative images showing differentiation morphology of SK-N-BE(2) cells. Scale bar = 50 µm. Bar graph indicates percentage of differentiated cells (n = 6, mean ± s.e.m.). **F** Immunoblotting for neuronal differentiation markers (RET, DLG2) in SK-N-BE(2) cells (n = 3). GAPDH was employed as immunoblotting loading control. Immunoblot experiments were performed in biologically independent triplicates. Quantification was performed with ImageJ and analyzed by Student *t* test (unpaired, two-tailed). *p* values are indicated (****P < 0.0001, ***0.0001 < *P* < 0.001, **0.001 < *P* < 0.01, *0.01 < *P* < 0.05, ns *P* ≥ 0.05); exact *P*-values are shown in Supplementary Fig. 3 and Supplementary Table 1.

ALK inhibition results in reduced proliferation of ALK-driven NB cells, while also resulting in ubiquitination of SLC3A2 and subsequent degradation. We therefore asked whether loss of SLC3A2 affects NB cell proliferation. We observed that siRNA-mediated knockdown of SLC3A2 over time reduced the proliferation rate in CLB-BAR, CLB-GE and CLB-GAR ALK-addicted NB cell lines with two independent siRNAs targeting SLC3A2 (Fig. 3A). Immunoblotting analysis confirmed a reduction in SLC3A2 protein levels on siRNA knockdown, together with a decrease in signaling activity, evidenced by decreased AKT and ERK phosphorylation compared with controls (Fig. 3B, Sup. Fig. 3A). Characterization of the effect of siRNA knockdown on cell cycle progression revealed accumulation in the sub-G1 phase after SLC3A2 knockdown in all cell lines (Fig. 3C). This was accompanied by elevated levels of apoptotic protein markers, such as cleaved PARP (PARP*) and γH2A.X (Fig. 3B, Sup. Fig. 3B). Thus, knockdown of SLC3A2 results in decreased proliferation, cell cycle regulation defects and induction of DNA-damage in NB cells.

### Knockdown of SLC3A2 promotes retinoic acid-induced neuronal differentiation

Previous studies have reported that knockdown of SLC3A2 in iPSCs promotes neuronal differentiation by neuronal induction medium [48], leading us to examine whether SLC3A2 knockdown might promote NB cell differentiation. Indeed, siRNA targeting of SLC3A2 resulted in increased neurite outgrowth and differentiation of CLB-GE cells (Fig. 3D). We further investigated the effect of SLC3A2 on neuronal differentiation in a second NB cell line, SK-N-BE (2) (*ALK-WT, 2p-gain* and *MYCN amp*.), in the presence or absence of retinoic acid (RA). While RA treatment alone induced neuronal differentiation in 32% of SK-N-BE (2) cells, knockdown of SLC3A2 further induced differentiation to 49% (Fig. 3E). Further, the expression of the established differentiation markers RET and DLG2 were upregulated (Fig. 3F, Sup. Fig. 3C).

### Knockdown of SLC3A2 decreased intracellular polyamine and branched-chain amino acid concentrations

**Fig. 4:**
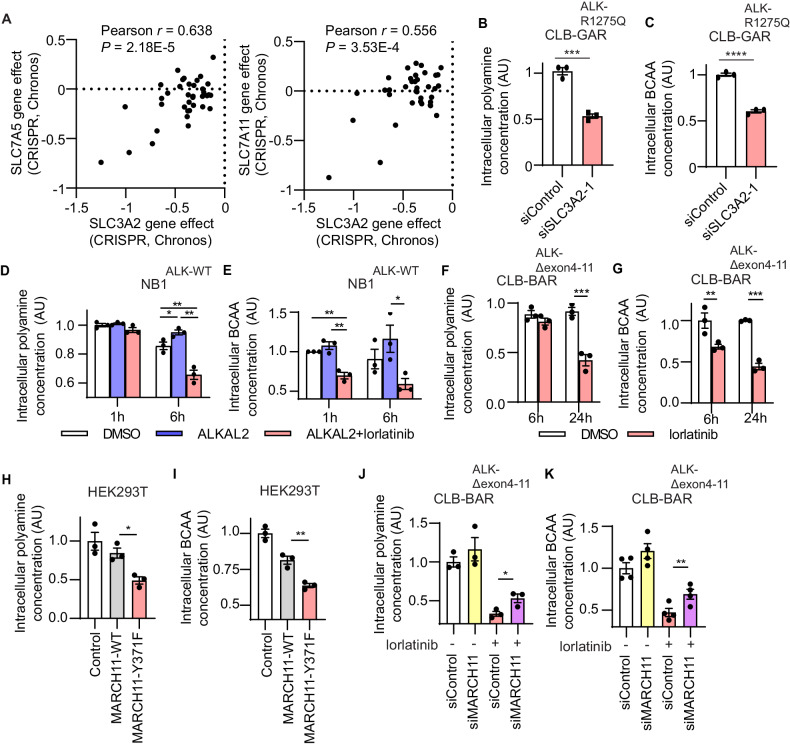
Knockdown of SLC3A2 decreases intracellular polyamine and branched-chain amino acid concentration. **A** Gene dependency effects (CRISPR, Chronos) of SLC3A2 with either SLC7A5 or SLC7A11 in NB cell lines (n = 37) from DepMap (https://depmap.org/portal/). **B** CLB-GAR cells were treated with either SLC3A2-targeting or control siRNAs. (**B**) Intracellular polyamine and (**C**) branched-chain amino acids (BCAA) concentrations were determined relative to controls (n = 3, mean ± s.e.m.). Intracellular polyamine (**D**) and BCAA (**E**) concentrations were determined at 0, 1 and 6 h of NB1 cell treatment with ALKAL2 (1 µg/ml) and lorlatinib (0 or 30 nM) (n = 3, mean ± s.e.m.). Intracellular polyamine (**F**) and BCAA (**G**) concentrations were determined at 0, 6 and 24 h of CLB-BAR cell treatment with lorlatinib (0 or 30 nM) (n = 3, mean ± s.e.m.). **H**, **I** Intracellular polyamine (**H**) and BCAA (**I**) concentrations were determined at 72 h after transection of HEK293T cells with either vector control, MARCH11-WT, or MARCH11-Y371F (n = 3, mean ± s.e.m.). **J, K** CLB-BAR cells were transfected with either control siRNA or MARCH11-targeting pool siRNAs for 24 h and lorlatinib (30 nM) added for additional 24 h. Intracellular polyamine (**J**) and BCAA (**K**) concentrations were determined (n = 3, mean ± s.e.m.). Results were analyzed by Student *t* test (unpaired, two-tailed). *p*-values are indicated (****P < 0.0001, ***0.0001 < *P* < 0.001, **0.001 < *P* < 0.01, *0.01 < *P* < 0.05, ns *P* ≥ 0.05); exact *P* values are shown in Supplementary Table 1.

We further investigated NB cell growth dependency on *SLC3A2* using the CRISPR-based gene effect results on DepMap portal (https://depmap.org/portal/). All NB cells investigated (n = 37) exhibited decreased cell growth after *SLC3A2* CRISPR knockout (Fig. 4A). Since SLC7A5 and SLC7A11 exist as heterodimeric membrane protein complexes with SLC3A2 (Sup Fig. 1C), we also investigated the growth dependency of *SLC7A5* and *SLC7A11* in NB cells and a potential correlation with *SLC3A2* dependency. The results showed that NB cells which depend on either SLC7A5 or SLC7A11 for cell growth have higher dependency on SLC3A2 for growth (Fig. 4A), consistent with previous findings that SLC7A5 and SLC7A11 require SLC3A2 for protein transport to the plasma membrane [42–44]. SLC3A2 is not only involved in the polyamine transport pathway in NB cells but also forms system L transporter complexes with SLC7A5 for essential branched chain amino acid (BCAAs; leucine, isoleucine, and valine) uptake [4, 49]. Consistent with the functions of SLC3A2, we detected decreased levels of intracellular polyamines and BCAA in NB cells on SLC3A2 knockdown (Fig. 4B, C). Since SLC3A2 is degraded upon lorlatinib treatment in ALK-addicted NB cells (Fig. 1C, D), we investigated whether intracellular polyamine and BCAA concentrations were affected in response to ALK inhibition. Both intracellular polyamine and BCAA concentrations were significantly decreased in NB1 and CLB-BAR cells after lorlatinib treatment (Fig. 4D–G).

To probe the functional significance of MARCH11 and its specific tyrosine phosphorylation site, we evaluated intracellular polyamine and BCAA concentrations in HEK293T cells overexpressing either MARCH11-WT or the MARCH11-Y371F mutant variant and CLB-BAR cells treated with MARCH11 siRNA or/and lorlatinib. In HEK293T cells, consistent with decreased SLC3A2 and SLC7A5 protein levels (Fig. 2H), intracellular polyamine and BCAA levels were found to be decreased in cells overexpressing the Y371F MARCH11 mutant compared to either those expressing wild-type MARCH11 or vector controls (Fig. 4H, I). Moreover, intracellular BCAA and polyamine levels were found to be significantly increased in lorlatinib treated MARCH11 knockdown CLB-BAR cells compared to controls (Fig. 4J, K), accompanied by elevated SLC3A2 and SLC7A5 protein levels (Fig. 2I). Together, these data suggest that MARCH11 and SLC3A2 are important in the regulation of intracellular polyamine and branched chain amino acid levels downstream of ALK in NB cells.

### ALK and SLC3A2 inhibitors synergistically inhibit NB cell growth

**Fig. 5:**
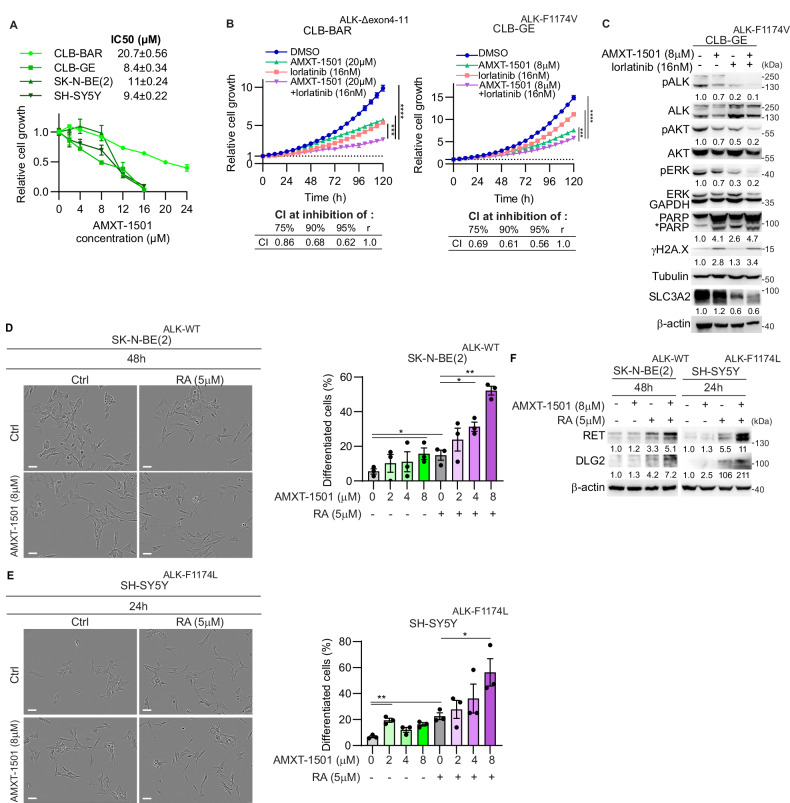
Combined lorlatinib and AMXT-1501 treatment synergistically inhibits cell growth in ALK-driven NB and increases retinoic acid-induced neuronal differentiation. **A** IC50s of AMXT-1501 in CLB-BAR, CLB-GE, SK-N-BE(2) and SH-SY5Y cells after 5 d treatment (n = 3, mean ± s.e.m.). **B** CLB-BAR and CLB-GE cells were treated with lorlatinib and AMXT-1501 at the indicated concentrations and cell growth monitored by Incucyte every 6 h over a total of 120 h (n = 3, mean ± s.e.m.). The combination index value was calculated based on Chou-Talalay method using CompuSyn software [70]. **C** Immunoblot analysis of ALK signaling and apoptosis markers in CLB-GE cells treated with AMXT-1501 (0 or 8 µM) and lorlatinib (0 or 16 nM) as indicated for 24 h (n = 3). Lysates were immunoblotted with anti-ALK, pY1278-ALK, pAKT(S473), AKT, pERK (T202/Y204), ERK, PARP (asterisk (*) marked cleaved PARP), γH2A.X, α-tubulin and SLC3A2 and β-actin. β-actin was employed as immunoblotting loading control. Representative images of differentiation morphology of SK-N-BE(2) (**D**) and SH-SY5Y (**E**) cells after AMXT-1501 (0 or 8 µM) and RA (0 or 5 µM) treatment as indicated for 48 h or 24 h. Scale bar = 50 µm. Bar graph indicates percentage of differentiated cells (n = 3, mean ± s.e.m.). **F** Immunoblot analysis of neuronal differentiation markers (RET and DLG2) in SK-N-BE(2) and SH-SY5Y cells treated with AMXT-1501 (0 or 8 µM) and RA (0 or 5 µM) for 48 h or 24 h (n = 3). β-actin was employed as immunoblotting loading control. Immunoblot experiments (**C** and **F**) were performed in biologically independent triplicates. Quantifications were performed with ImageJ and analyzed by *Student t*-test (unpaired, two-tailed). *p* values are indicated (****P < 0.0001, ***0.0001 < *P* < 0.001, **0.001 < *P* < 0.01, *0.01 < *P* < 0.05, ns *P* ≥ 0.05); exact *P* values are shown in Supplementary Fig. 4 and Supplementary Table 1.

Our observation of accumulation of cells in sub-G1 cell cycle arrest on siRNA knockdown of SLC3A2 is in agreement with earlier report employing the AMXT-1501 polyamine transport inhibitor in combination with alpha-difluoromethylornithine (DFMO), an ornithine decarboxylase inhibitor or cisplatin [50–52]. More lately it has been shown that AMXT-1501 enhances the cytotoxic effects of cisplatin in vitro and in vivo in aggressive head and neck squamous cell carcinoma cell lines [53]. To investigate AMXT-1501 in an NB setting, IC50s were determined in four NB cell lines including CLB-BAR, CLB-GE, and SH-SY5Y (*ALK-F1174L*) and SK-N-BE (2). Cells were treated for 5 d with serial dilutions of AMXT-1501 ranging from 2 to 24 µM (Fig. 5A) and relative cell growth was determined by cell confluence in a Sartorius Incucyte S3. AMXT-1501 exhibited cytotoxic activity against both ALK-addicted cell lines, with IC50 values of 8.4 µM for CLB-GE and 20.7 µM for CLB-BAR. A similar effect was observed in the non-ALK driven cell lines, 11 µM for SK-N-BE(2) and 9.4 µM for SH-SY5Y cells. To investigate whether the combination of ALK and SLC3A2 inhibition was synergistic, we treated CLB-BAR and CLB-GE cells with either AMXT-1501 alone or in combination with lorlatinib (16 nM) (Fig. 5B). Cells were treated with AMXT1501 at the estimated IC50 value (8 µM and 20 µM AMXT-1501 in CLB-GE and CLB-BAR cells, respectively). Combination treatment exhibited synergistic abrogation of growth in both cell lines (Fig. 5B). Synergy calculation with the Chou-Talalay index indicated a synergistic effect of AMXT-1501 and lorlatinib when combined (Fig. 5B). Analysis of lysates from treated cells showed that the AMXT-1501/lorlatinib combination synergistically suppressed SLC3A2 protein expression levels as well as ALK signaling output (measured as pALK, pAKT, and pERK), while simultaneously inducing apoptosis (cleaved PARP), and DNA damage (γH2A.X) (Fig. 5C, Sup Fig. 4A). As SLC3A2 knockdown promotes RA-induced neuronal differentiation (Fig. 3E), we further investigated whether SLC3A2 inhibition by AMXT-1501 treatment was able to potentiate RA-induced neuronal differentiation. Treatment with AMXT-1501 in the presence of RA revealed a dose-dependent increase in cell differentiation in both SK-N-BE (2) and SH-SY5Y cells (Fig. 5D, E, Sup Fig. 4B, C) accompanied by elevated expression of RET and DLG2 differentiation markers (Fig. 5F, Sup Fig. 4D). Neurite length and branch points normalized to cell-body cluster area were significantly increased by AMXT-1501 in the presence of RA, compared to RA-treated controls (Sup Fig. 4B, C).

### ALK signaling harnesses amino acid transporter systems for more efficient nutrient uptake

**Fig. 6:**
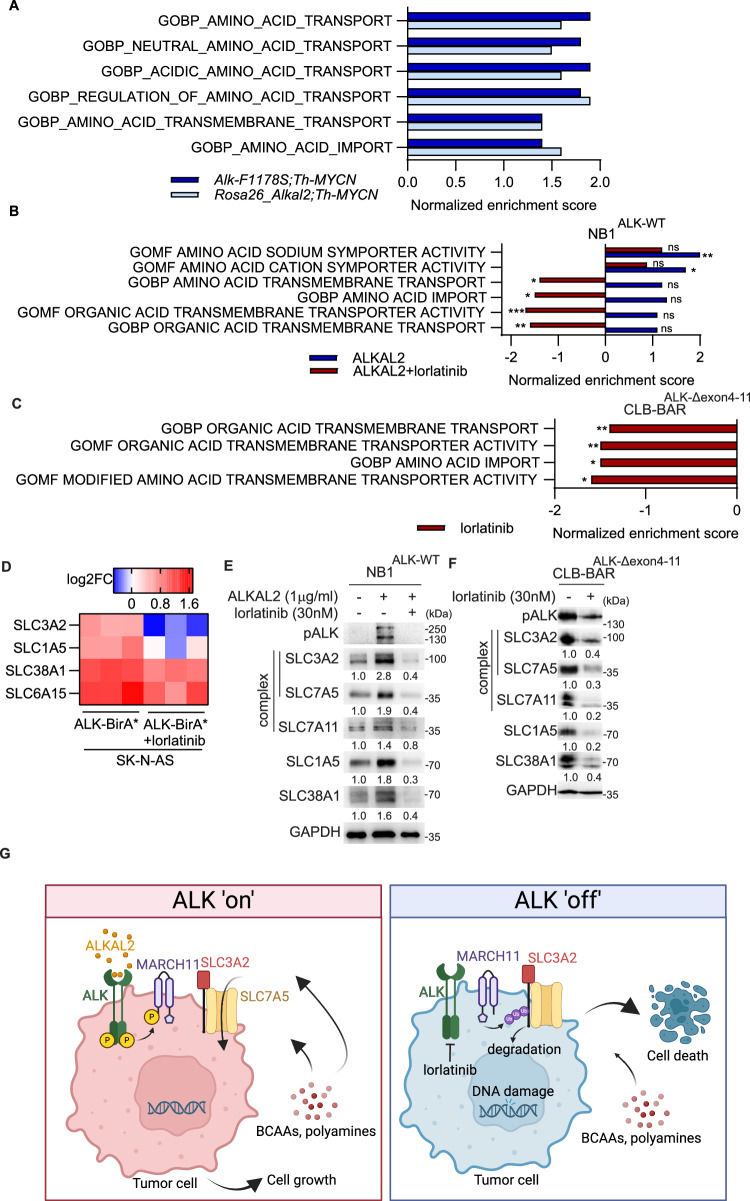
ALK signaling upregulates amino acid transporter systems. Gene oncology GSEA analysis of RNA-seq datasets. **A** NB tumors from either *Alk-F1178S;Th-MYCN* mice or *Rosa26_Alkal2;Th-MYCN* mice compared to *Th-MYCN* mice. **B** NB1 cells treated with either ALKAL2 or/and lorlatinib for 24 h. **C** CLB-BAR cells treated with lorlatinib for 24 h compared to DMSO treated control. **D** Heat map indicating biotinylation of SLC1A5, SLC3A2, SLC6A15, SLC38A1 in SK-N-AS.ALK-BirA* expressing cells compared to BirA* controls. Columns represent three biological replicates, data from [41]. Immunoblotting for anti-pALK (Y1278), SLC1A5, SLC3A2, SLC7A5, SLC7A11, SLC38A1 and GAPDH in NB1 cells treated with ALKAL2 (1 µg/ml) and lorlatinib (0 or 30 nM) for 24 h (**E**) and CLB-BAR cells treated with lorlatinib (0, 30 nM) for 24 h (**F**). **G** Schematic visualization of the regulation of SLC3A2 downstream of ALK in NB cells (created with BioRender.com). Immunoblot experiments were performed in biologically independent triplicates. Quantifications were performed with ImageJ and analyzed by Student *t* test (unpaired, two-tailed). *p* values are indicated (****P < 0.0001, ***0.0001 < *P* < 0.001, **0.001 < *P* < 0.01, *0.01 < *P* < 0.05, ns *P* ≥ 0.05); exact *P* values are shown in Supplementary Fig. 5 and Supplementary Table 1.

We examined the effect of ALK signaling on amino acid transport systems in our previous RNA-seq data derived from *Alk-F1178S;Th-MYCN, Rosa26_Alkal2;Th-MYCN* or *Th-MYCN* NB tumors [54] and NB cells treated with ALKAL2 or/and ALK inhibitor [38, 54]. GSEA Gene Ontology analysis identified a strong enrichment of biological pathways related to amino acid transport in NB tumors from both ALK gain-of-function mice (*Alk-F1178S;Th-MYCN*, NES = 1.9, *P* = 5.8E-7) and ALKAL2 overexpressing mice (*Rosa26_Alkal2;Th-MYCN*, NES = 1.6, *P* = 3.5E-4) compared to *Th-MYCN* mice (Fig. 6A, Sup. Fig. 5A, Sup. Table). In line with our observations in mouse tumors, stimulation of ALK signaling with ALKAL2 ligand in NB1 cells, resulted in an enrichment of amino acid sodium symporter activity (NES = 2, *P* = 4.5E-3) as well as cation symporter activity (NES = 1.7, *P* = 1.5E-2) (Fig. 6B, Sup. Table). Upon ALK inhibitor treatment, biological pathways related to organic acid transmembrane transport (NES = −1.6, *P* = 1.5E-3), organic acid transmembrane transporter activity (NES = −1.7, *P* = 2.7E-4), amino acid import (NES = −1.5, *P* = 2E-2), and amino acid transmembrane transport (NES = −1.4, *P* = 2.7E-2) were suppressed in NB1 cells (Fig. 6B, Sup. Fig. 5B, Sup. Table). Molecular functions related to amino acid transport were also down-regulated in ALK mutant-driven CLB-BAR cells including organic acid transmembrane transport (NES = -1.4, *P* = 4.9E-3), organic acid transmembrane transporter activity (NES = −1.5, *P* = 1.3E-3), amino acid import (NES = −1.5, *P* = 1.8E-2) and modified amino acid transmembrane transporter activity (NES = −1.6, *P* = 1.7E-2) (Fig. 6C, Sup. Fig. 5C, Sup. Table).

We next examined the effect of ALK signaling on those SLC amino acid transporters identified in our earlier BioID-based in vivo proximity labeling study in SK-N-AS.ALK-BirA* cells, where SLC1A5, SLC38A1, and SLC6A15 were also identified as potential interacting partners with ALK in addition to SLC3A2 (Fig. 6D) [41]. To further validate the effect of ALK activity on SLC protein levels, NB1 and CLB-BAR were treated with either ALKAL2 ligand or/and lorlatinib. Activation of ALK with ALKAL2 ligand resulted in a robust upregulation of SLC3A2, SLC7A5 and SLC7A11 (both interaction partners of SLC3A2), SLC1A5, and SLC38A1 protein levels. In keeping with an important role of ALK activity, this effect was blocked by the addition of lorlatinib (Fig. 6E, Sup. Fig. 5D). Treatment of CLB-BAR cells with lorlatinib further confirmed that ALK signaling regulates SLC1A5, SLC3A2, SLC7A5, SLC7A11, SLC38A1 protein levels in ALK-addicted NB cell lines (Fig. 6F, Sup. Figure 5E). Taken together, these results suggest that ALK signaling influences protein levels of multiple SLC amino acid transporters supporting increased nutrient uptake and NB cell survival (Fig. 6G).

## Discussion

Disruptions in polyamine metabolism are commonly observed in human cancer, where elevated polyamine concentrations are thought to play a crucial role in promoting cell proliferation and the development of tumors [55, 56]. The ornithine decarboxylase (ODC1) inhibitor, difluoromethylornithine (DFMO), results in in polyamine depletion and is cytostatic in mammalian cells with modest therapeutic effect, therefore research has focused on DFMO in combination with other drugs [57–59] as well as with the polyamine transport inhibitor AMXT-1501 [4, 50–52, 60].

The childhood cancer NB, is a heterogeneous disease that exhibits *ALK* mutations in up to 10% of primary NB, and at higher levels on relapse [19–23]. This has led to ALK inhibition being employed clinically for ALK-positive NB patients [61, 62]. While a combination of DFMO and AMXT-1501 has been explored in NB [4, 50], no study has yet investigated a potential effect of polyamine metabolism disruption in combination with ALK inhibition.

Here we have investigated an interaction between ALK and SLC3A2, which was reported in a proximity labeling BioID analysis in NB cell lines [41]. This novel interaction between an RTK and a protein involved in polyamine transport was unexpected, prompting us to explore the functional relevance of the ALK:SLC3A2 interaction in an NB context. SLC3A2 has been reported to be present in complex with SLC7A5 and SLC7A11 [42–44], and in keeping with a functional relevance in NB, SLC3A2 and SLC7A5 are highly prognostic of poor outcome in the R2 dataset of NB (KOCAK dataset, n = 649) [4]. SLC3A2 has been shown to be one of the proteins involved in the polyamine transport pathway in NB, and in combination with DFMO (ODC1 inhibitor), was shown to inhibit tumor development in *TH-MYCN* transgenic mice [4]. However, to the best of our knowledge, no functional connection between ALK and SLC3A2 has been reported.

Here we show that ALK and SLC3A2 interact. Moreover, stimulation with the ALK ligand ALKAL2 resulted in a rapid upregulation of SLC3A2 protein levels, while inhibition of ALK leads to loss of SLC3A2 protein. This dramatic response is a consequence of ALK regulation of SLC3A2 ubiquitination and subsequent degradation, where ALK inhibition results in increased ubiquitination and loss of SLC3A2. Post-translational modifications (PTMs) such as phosphorylation, ubiquitination, glycosylation and lipid modifications have been shown to regulate the expression of SLC transporters [42, 63]. These covalent additions often occur as a result of signaling cascades [63]. For example, SLC22A2 is regulated by a tyrosine phosphorylation switch via the Yes1 Src family kinase and dephosphorylation of SLC22A2 diminishes transport without altering its membrane expression [63]. Other transporters which are also regulated by Src family kinases through tyrosine residue phosphorylation include the serotonin transporter (SERT; SLC6A4) [64, 65] and the apical sodium-dependent bile acid transporter (ASBT; SLC10A2) [66]. SLC transporters are also regulated by ubiquitination, for example, by the MARCH8 ubiquitin ligase that has been shown to ubiquitinate SLC3A2 and promote its degradation [45]. Interestingly, the MARCH ubiquitin ligases are themselves regulated by phosphorylation, e.g., MARCH3 has been reported to be tyrosine phosphorylated downstream of the TYRO3 RTK, a modification that inhibits MARCH3 ubiquitin ligase activity [46, 47].

While ALK signaling via the Cbl family of ubiquitin ligases is important for negative feedback regulation [32], a role for the MARCH family ubiquitin ligases in ALK signaling has not yet been reported in NB. Of the 11 MARCH protein family members, we observed that several were detected at the mRNA level in public NB cell line data (https://depmap.org/portal/). Of these, *MARCH11* expression was enriched in NB. There are only a few reports regarding MARCH11 in the literature, however, it has previously been reported as a ubiquitin ligase with role in sorting in the trans-golgi network [67]. Moreover, several phosphoproteomics studies of ALK signaling, including our own reports, contain MARCH11 phosphorylation sites that are sensitive to ALK inhibition [38, 68], suggesting that ALK may indeed regulate SLC transporters via the MARCH ubiquitin ligases to promote NB cell growth. Our data identify an ALK/MARCH11/SLC3A2 regulatory module and suggest that ALK inhibition results in MARCH11 dephosphorylation, which leads to activation of MARCH11 ligase activity and subsequent ubiquitination of SLC3A2. This scenario is reminiscent of the TYRO3 regulation of MARCH3, and to our knowledge, is the first report of a complex containing ALK, a MARCH family E3 ligase (MARCH11) and an amino acid transporter (SLC3A2) where the stability of the amino acid transporter is regulated downstream of ALK. In agreement with a role for ALK signaling in regulating SLC3A2, we observed decreased intracellular polyamine and branched chain amino acid concentrations upon treatment with ALK inhibitors in NB cells.

Inhibition of ALK activity has been shown in many experimental systems to reduce ALK-driven NB cell and tumor growth [24, 69]. Our data here shows that loss of SLC3A2 also reduces the proliferation rate of ALK-driven NB cell lines. Combination of AMXT-1501 together with lorlatinib synergistically reduced growth in ALK-driven NB cell lines. We also observed that either knockdown of SLC3A2 or treatment with AMXT-1501 potentiated retinoid acid induced neuronal differentiation in NB cells, in agreement with earlier reports that SLC3A2 inhibition in iPSCs promotes neuronal differentiation [48]. Studies targeting polyamine metabolism in NB have shown that combination of DFMO (ODC1 inhibitor) with the polyamine transport inhibitor, AMXT-1501, synergistically reduces cell viability in NB cells [50]. In a recent study, Haber and coworkers showed that MYCN regulates expression of the entire polyamine pathway in high-risk NB through increased polyamine synthesis, decreased polyamine catabolism, and increased polyamine uptake [4]. In this work, an AMXT-1501/DFMO combination treatment delayed tumor development in a *Th-MYCN*-driven NB mouse model [4]. Our data suggest that AMXT-1501 in combination with an ALK inhibitor may be effective in ALK-driven NB models.

In this work we have focused on SLC3A2 and ALK, however, our original BioID analysis of the ALK interactome in NB cells also identified a number of other transporters, such as SLC1A5, SLC38A1 and SLC6A15 as potential ALK partners [41]. Data mining of earlier experiments in NB cells treated with either ALK ligand or ALK inhibitors revealed an ALK-dependent modulation of other transporters at the protein level in ALK-driven NB cell lines. Thus, ALK signaling has a wider influence on the expression and regulation of the SLC amino acid transporters, that likely support increased nutrient uptake and promote NB growth and survival. While we have elucidated an underlying molecular mechanism for the regulation of SLC3A2 by ALK that involves the MARCH11 E3 ligase, further work will be required to understand in detail how other SLC transporters are regulated.

Taken together, our results show that ALK interacts with and via the MARCH11 ubiquitin ligase regulates SLC3A2 protein stability in NB cells (Fig. 6G). The synergistic effect of combined ALK and polyamine transport inhibitors suggests that ALK-driven NB cells are highly sensitive to dual inhibition of polyamine metabolism and ALK, with the novel ALK/MARCH11/SLC3A2 regulatory module identified here ultimately regulating polyamine transport and supporting oncogenic growth downstream of ALK in NB cells.

## Methods

### Antibodies

Primary antibodies against human SLC3A2 (#81977, was employed for immunoblotting, 1:2000; #13180, was used for immunoprecipitation), ALK (#3633, 1:2000), pALK (Y1278, #6941, 1:1000), ubiquitin (#3936, 1:1000), phospho-Tyrosine (p-Y-1000, #8954, 1:1000), pAKT (S473,#4060, 1:4000), AKT (#9272), pERK1/2 (Y204/T202, #4377, 1;2000), PARP (#9542, 1:1000), γH2A.X (Ser139, #9718, 1:1000), RET (#14556, 1:2000), DLG2 (#19046, 1:1000), SLC1A5 (ASCT2, #8057, 1:2000), SLC7A5 (LAT1, #32683, 1:1000), SLC7A11 (xCT, #12691, 1:1000), SLC38A1 (SNAT1, #36057, 1:2000), β-actin (#4970, 1:10000), GAPDH (#5174, 1:10,000) and α-tubulin (#2125, 1:10,000) were obtained from Cell Signaling Technology. Antibodies to MARCH11 (#AP13811B, 1:1000) were purchased from Abcepta, anti-FLAG (#F1804, 1:3000) was from Sigma, and panERK (#610124, 1:5000) was from BD Transduction Laboratories. Horseradish peroxidase-conjugated secondary antibodies, goat anti-mouse immunoglobulin G (IgG) (# 32230), and goat anti-rabbit IgG (# 32260, 1:5000) were purchased from Thermo Fisher Scientific.

### Cell culture

NB1 (wild-type ALK), CLB-BAR (gain of function, Δexon4-11 truncated ALK), CLB-GE (gain of function, ALK-F1174V mutation), CLB-GAR (gain of function, ALK-R1275Q mutation, 11q-deletion), SK-N-BE(2) (wild-type ALK, 2p-gain, MYCN amplified), SH-SY5Y (gain of function, ALK-F1174V mutation) and HEK293T were employed in this study. CLB-BAR, CLB-GE and CLB-GAR were obtained from The Centre Leon Bernard, France under MTA. SK-N-BE (2), SH-SY5Y and HEK293T were purchased from ATCC. All cell lines were confirmed to be free of mycoplasma contamination. All NB cell lines were cultured in complete media (RPMI-1640 supplemented with 10% FBS and 1% penicillin/streptomycin, Gibco) on plates precoated with 0.4% solution of type I bovine collagen solution. HEK293T cells were cultured in DMEM supplemented with 10% FBS and 1% penicillin/streptomycin (Gibco). ALK was stimulated with ALKAL2 (1 µg/ml) or/and inhibited by lorlatinib (30 nM, Selleckchem) for 0, 1, 6, 24 h in NB1 cells (n = 3 biologically independent experiments). CLB-BAR, CLB-GE and CLB-GAR cells were treated with lorlatinib (30 nM) for 0, 6, 24 h (n  =  3 biologically independent experiments). For cycloheximide chase assays, CLB-BAR, CLB-GE and CLB-GAR cells were treated with cycloheximide (50 µg/ml, Sigma-aldrich) in the presence or absence of lorlatinib (30 nM) for 0, 1, 3, 5, 7 h prior to lysate collection and immunoblot analysis (n = 3 biologically independent experiments). For MG132 assays, NB1 and CLB-GE cells were treated with MG132 (10 µM) in the presence or absence of ALKAL2 (1 µg/ml) or/and lorlatinib (30 nM) for 0, 24 h (n = 3 biologically independent experiments).

### Co-immunoprecipitation assays

NB1 cells were treated with ALKAL2 (1 µg/ml) or/and lorlatinib (30 nM) or/and MG132 (10 µM), CLB-GE and CLB-BAR cells were treated with lorlatinib (30 nM) for 24 h prior to lysis with IP buffer (150 mM NaCl, 50 mM Tris-HCl, 1% Triton X-100, 1% glycerol) containing protease inhibitor cocktail (#04693132001, Roche). Either 3 µg anti-ALK (#3633, CST), anti-SLC3A2 (#13180, CST), anti-phospho-Tyrosine (p-Y-1000, #8954, CST) or isotype control (#AB-105-C, R&D systems) antibody was added to 300 µg of cell lysate for overnight rotation at 4 °C, followed by incubation with 25 µl Protein G Sepharose 4 Fast Flow for an additional 2 h. Samples were washed twice with high salt IP buffer (500 mM NaCl, 50 mM Tris-HCl, 1% Triton X-100, 1% glycerol) and then washed once with IP buffer (150 mM NaCl, 50 mM Tris-HCl, 1% Triton X-100, 1% glycerol). Proteins were eluted in 2X Laemmli buffer with heating for 10 min at 95 °C. Samples were then subjected to SDS-PAGE for immunoblot analysis and 5 µg lysate loaded as input (n = 3 biologically independent experiments).

### MARCH11 overexpression and knockdown

The *MARCH11* cDNA ORF clone (CloneID #OHu01478) generated from pcDNA3.1-C-(k)DYK vector was purchased from Genscript (MARCH11-WT). A mutation was introduced at amino acid Y371, substituting Y to F (MARCH11-Y371F), to produce a non-phosphorylatable form. For MARCH11 overexpression, HEK293T cells were transfected with 2 µg of pcDNA3.1, MARCH11-WT, or MARCH11-Y371F plasmids by X-tremeGENE™ HP DNA Transfection Reagent (Roche) for 72 h. For MARCH11 knockdown, CLB-BAR cells were transfected with 50 nM of pool siRNAs targeting MARCH11 (ON-TARGETplus Human MARCH11 siRNA SMARTpool, #L-033939-02-0005, Horizon Discovery) by Lipofectamine^TM^ RNAiMAX Transfection Reagent (Invitrogen) for 48 h. Lorlatinib was added to the media 24 h after transfection. Samples were then subjected to immunoblot analysis and intracellular polyamine and branched-chain amino acid concentration determination (n = 3 biologically independent experiments).

### Cell proliferation and cell cycle assays upon siRNA treatment

CLB-BAR, CLB-GE and CLB-GAR cells were transfected with siRNAs targeting SLC3A2 (Silencer® select s12944 or s12945, Thermo Fisher Scientific) by Lipofectamine^TM^ RNAiMAX Transfection Reagent (Invitrogen). Cell growth was monitored live at 37 °C using a Sartorius Incucyte S3 (Essen BioScience) by scanning the cells at 6 h intervals for 72 to 120 h. Cell confluency was determined using Sartorius Incucyte S3 software (Essen Bioscience) (n = 3 biologically independent experiments, mean ± s.e.m.). For cell cycle analysis, cells were transfected with siRNAs for 72 h, after which they were treated according to a 2-step cell cycle assay protocol and analyzed using a NucleoCounter® NC-3000™ system (Chemometec) (n = 3 biologically independent experiments, mean ± s.e.m.). For immunoblot analysis, protein samples from CLB-BAR, CLB-GE and CLB-GAR cells were collected at 72 h after siRNA transfections and immunoblotted against primary antibodies targeting SLC3A2, pAKT, AKT, pERK, ERK, GAPDH, PARP/cleaved PARP (PARP*), γH2A.X and α-tubulin (n = 3 biologically independent experiments). Quantifications were performed using ImageJ and normalized first to internal controls (GAPDH or α-tubulin) and then to the group transfected with negative control siRNA.

### Cell differentiation assay upon siRNA treatment

SK-N-BE(2) cells were transfected with siRNAs targeting SLC3A2 (Silencer® select s12944 or s12945, Thermo Fisher Scientific) by Lipofectamine^TM^ RNAiMAX Transfection Reagent (Invitrogen) for 24 h and then treated with retinoic acid (RA, 5 µM) for another 48 h. Cell differentiation was recorded in an Incucyte S3 (Essen BioScience). A total of 108 random fields (18 random fields from 6 independent biological replicates) were analyzed by NeuroTrack software (Essen BioScience). Differentiated cells were defined as a cell with neurite projecting at least 1.5 times the cell body length (n = 6 biologically independent experiments, mean ± s.e.m.). For immunoblotting, primary antibodies against RET, DLG2, SLC3A2 and GAPDH were employed. Quantification of RET and DLG2 protein levels was performed with ImageJ and normalized first to GAPDH and then to the group transfected with negative control siRNA and without RA treatment.

### Intracellular polyamine and branched chain amino acid concentration determination

CLB-GAR cells were transfected with 50 nM of siRNAs targeting SLC3A2 (Silencer® select s12944, Thermo Fisher Scientific) by Lipofectamine^TM^ RNAiMAX Transfection Reagent (Invitrogen) for 48 h. For ALK signaling, NB1 cells were treated with ALKAL2 (1 µg/ml) or/and lorlatinib (30 nM) for 0, 1, 6 h and CLB-BAR cells were treated with lorlatinib (30 nM) for 0, 6, 24 h. Intracellular polyamine and branched chain amino acid concentrations were determined with a Total Polyamine Assay Kit (#ab239728, abcam) and a Branched Chain Amino Acid Assay Kit (#ab83374, abcam) respectively and normalized first to the viable cell numbers used to obtain assay lysates and then to the group transfected with negative control siRNA or DMSO group at 0 h time point (n = 3 biologically independent experiments, mean ± s.e.m.).

### Polyamine transport inhibitor treatment

CLB-BAR, CLB-GE, SK-N-BE(2) and SH-SY5Y cells were treated with AMXT-1501 (0, 2, 4, 8, 12, 16, 20, and 24 µM, MedChemExpress). Cell growth was monitored with a Sartorius Incucyte S3 (Essen BioScience) and fold change in cell confluency determined using Sartorius Incucyte S3 software (Essen Bioscience) (n = 3 biologically independent experiments, mean ± s.e.m.). For drug synergy investigation, CLB-BAR cells were treated with AMXT-1501 (0 or 20 µM) and lorlatinib (0 or 16 nM). CLB-GE cells were treated with AMXT-1501 (0 or 8 µM) and lorlatinib (0 or 16 nM). Cell growth was monitored as previously described (n = 3 biologically independent experiments, mean ± s.e.m.). To determine synergy for the AMXT-1501/lorlatinib combination treatment, combination index (CI) values were calculated based on the results after 5 d treatment using the CompuSyn software based on the Chou and Talalay method [70]. For immunoblot analyses, CLB-GE cells were treated with AMXT-1501 (0 or 8 µM) and lorlatinib (0 or 16 nM) for 24 h (n = 3 biologically independent experiments). Primary antibodies recognizing SLC3A2, pALK, ALK, pAKT, AKT, pERK, ERK, GAPDH, PARP/cleaved PARP (PARP*), γH2A.X, β-actin and α-tubulin were used. For neurite outgrowth assays, SK-N-BE(2) and SH-SY5Y cells were treated with AMXT-1501 (0, 2, 4, and 8 µM) and retinoic acid (RA, 0 or 5 µM) for 48 h and 24 h, respectively (n = 3 biologically independent experiments, mean ± s.e.m.). Percentage of differentiated cells and RET/DLG2 protein levels were determined as described above. Neurite length (mm) and branch points normalized to cell body area (mm^2^) were analyzed by NeuroTrack software (Essen BioScience).

### Gene sets and gene set enrichment analyses

RNA expression level and gene effect scores (dependency scores, CRISPR, Chronos) of ALK, SLC3A2, SLC7A5, SLC7A11, MARCH protein family members in NB cell lines were derived from DepMap portal [71, 72](https://depmap.org/portal/). After normalization, nonessential genes have median score of 0 and predefined common essentials have a median score of -1.

RNA-Seq data from [1] neuroblastoma tissues from transgenic mice with *Th-MYCN, Alk-F1178S;Th-MYCN*, or *Rosa26_Alkal2;Th-MYCN* background [54], (2) NB1 cells treated with ALKAL2 or/and lorlatinib for 24 h [54] and (3) CLB-BAR and CLB-GE cells treated with lorlatinib for 24 h [38] were analyzed. Preranked gene set enrichment analyses (GSEA) were performed using the R fgsea package (fgseaMultilevel function, default parameters) with ranking based on the DEseq2 statistic. Mouse and human gene sets were downloaded from the Molecular Signatures Database (MSigDB) v2023.1.

### Statistical analysis

Experiments were conducted at least in triplicate using biologically independent samples, and results presented as mean ± standard error of the mean (SEM). Statistical significance was assessed using Student *t* test (unpaired, two-tailed) in GraphPad Prism 9 software and GP formatting was used to show *p* values (****P < 0.0001, ***0.0001 < *P* < 0.001, **0.001 < *P* < 0.01, *0.01 < *P* < 0.05, ns *P* ≥ 0.05). For RNA-Seq data and preranked Gene Set Enrichment Analysis (GSEA), R statistical package (version 4.0) and CLEAN (Cell Line Explorer web Application of Neuroblastoma data; https://ccgg.ugent.be/shiny/clean/) [73] was utilized. Specific statistical tests performed and exact *p* values are detailed in the respective sections, figure legends, and supplementary figures and table.

### Supplementary information

Supplementary Figures and table legends

Supplementary Figures

Supplementary Tables

Uncropped western blots

## Data Availability

The datasets generated during and/or analyzed during the current study are available in the CLEAN (Cell Line Explorer web Application of Neuroblastoma data; https://ccgg.ugent.be/shiny/clean/).
