# Supplementary material for: Anaplastic Lymphoma Kinase signaling stabilizes SLC3A2 expression via MARCH11 to promote neuroblastoma cell growth
Source: Cell Death Differ. 2024 Jun 10;31(7):910–23. doi: 10.1038/s41418-024-01319-0 (PMC11239919; doi:10.1038/s41418-024-01319-0)
Supplement: Supplementary file 1 — Supplementary Figures and table legends [file 41418_2024_1319_MOESM1_ESM.docx]

**Supplementary Figures**

**Supplementary Figure 1. (A)** Additional immunoblot replicates (two independent) are shown for NB1 cells treated with ALKAL2 (1 µg/ml) and lorlatinib (0 or 30 nM) for 24 h. Lysates were subjected to immunoprecipitation with anti-ALK or anti-SLC3A2 antibodies followed by immunoblotting for ALK and SLC3A2. Anti-rabbit IgG was employed as negative control. **(B)** Additional immunoblot replicates (two independent) are shown for CLB-GE cells treated with lorlatinib (0 or 30 nM) for 24 h. Lysates were subjected to immunoprecipitation with anti-SLC3A2 antibodies followed by immunoblotting for ALK and SLC3A2. Anti-rabbit IgG was employed as negative control. **(C)** SLC3A2 forms a complex with SLC7A5 and with SLC7A11 in NB1 cells. **(D)** Additional immunoblot replicates (two independent) are shown for NB1 cells treated with ALKAL2 (1 µg/ml) and lorlatinib (0 or 30nM) for 0, 1 and 6 h. Lysates were immunoblotted for ALK, pY1278-ALK and SLC3A2. β-actin was employed as loading control. Quantification was performed with ImageJ and analyzed by *Student t*-test (unpaired, two-tailed). Exact *p*-values are indicated. **(E)** Additional immunoblot replicates (two independent) are shown for time-course of CLB-BAR, CLB-GE and CLB-GAR cells treated with lorlatinib (30 nM). Lysates were immunoblotted for ALK, pY1278-ALK and SLC3A2. β-actin was employed as loading control. Quantification was performed with ImageJ and analyzed by *Student t*-test (unpaired, two-tailed). Exact *p*-values are indicated. **(F)** Additional immunoblot replicates (two independent) are shown for CLB-BAR, CLB-GE, and CLB-GAR cells treated with CHX (50 µg/ml) and lorlatinib (0 or 30 nM) for the times indicated. SLC3A2 and β-actin protein levels were detected by immunoblotting and quantified by ImageJ software (n=3, mean ± s.e.m.). Exact *p*-values are indicated. **(G)** Additional immunoblot replicates (two independent) are shown for NB1 cells treated with ALKAL2 (1 µg/ml), lorlatinib (0 or 30 nM), and MG132 (0 or 10 µM) for 24 h. Lysates were immunoblotted for ALK, pY1278-ALK and SLC3A2. β-actin was employed as loading control. Quantification was performed with ImageJ and analyzed by *Student t*-test (unpaired, two-tailed). Exact *p*-values are indicated. **(H)** Additional immunoblot replicates (two independent) are shown for CLB-GE cells treated with lorlatinib (0 or 30 nM) and MG132 (0 or 10 µM) for 24 h. Lysates were immunoblotted for SLC3A2 and β-actin. β-actin was employed as loading control. Quantification was performed with ImageJ and analyzed by *Student t*-test (unpaired, two-tailed). Exact *p*-values are indicated.

**Supplementary Figure 2. (A)** Additional immunoblot replicates (two independent) are shown for NB1 cells treated with ALKAL2 (1 µg/ml), lorlatinib (0 or 30nM) and MG132 (10 µM) for 24 h. Lysates were subjected to immunoprecipitation with anti-SLC3A2 antibodies and immunoprecipitated complexes were immunoblotted for SLC3A2 and ubiquitin (Ub). Anti-rabbit IgG was employed as control for immunoprecipitation. Quantification was performed with ImageJ. **(B)** RNA expression levels of MARCH protein family members in NB cells. Data from Depmap portal (<https://depmap.org/portal/>). **(C-D)** Additional immunoblot replicates (one or two independent) are shown for CLB-BAR cells treated with lorlatinib (0 or 30 nM) for 6 h and lysates subjected to immunoprecipitation with anti-SLC3A2 or p-Y1000 antibodies. Immunoblotting was performed to detect ALK, MARCH11 and SLC3A2 (C), and phosphorylation of ALK and MARCH11 (D). IgG heavy chains are indicated with asterisk (*). **(E)** Additional immunoblot replicates (two independent) are shown for HEK293T cells lysed 72 h after transfection with either vector control, MARCH11-WT or MARCH11-Y371F. Lysates were immunoblotted for SLC3A2, SLC7A5, and Flag. Tubulin was employed as loading control. Quantification was performed with ImageJ and analyzed by *Student t*-test (unpaired, two-tailed). Exact *p*-values are indicated. **(F)** Additional immunoblot replicates (two independent) are shown for CLB-BAR cells treated with either control siRNA or MARCH11-targeting pool siRNAs for 24 h, followed by lorlatinib (30nM) treatment for an additional 24 h. Lysates were immunoblotted for pY1278-ALK, SLC3A2, SLC7A5, and MARCH11. Tubulin was employed as loading control. Quantification was performed with ImageJ and analyzed by *Student t*-test (unpaired, two-tailed). Exact *p*-values are indicated.

**Supplementary Figure 3. (A-B)** **(A)** Additional immunoblot analyses (in triplicate for two indepenant siRNAs targeting SCL3A2) are shown for CLB-BAR, CLB-GE, and CLB-GAR cells. Cells were treated with either control siRNA or SLC3A2-targeting siRNAs (2-1 and 2-2). Lysates collected at 72h after siRNA transfection were immunoblotted with anti-SLC3A2, β-actin, pAKT(S473), AKT, pERK (T202/Y204), ERK and GAPDH. GAPDH was employed as immunoblotting loading control. Quantification was performed with ImageJ and analyzed by *Student t*-test (unpaired, two-tailed). Exact *p*-values are indicated. **(B)** Additional immunoblot replicates (two independent) are shown for CLB-BAR, CLB-GE, and CLB-GAR cells treated with either control siRNA or SLC3A2-targeting siRNA. Lysates collected at 72h after siRNA transfection were immunoblotted with anti-PARP (asterisk (*) marked cleaved PARP), γH2A.X and α-tubulin. α-tubulin was employed as loading control. Quantification was performed with ImageJ and analyzed by *Student t*-test (unpaired, two-tailed). Exact *p*-values are indicated. **(C)** Additional immunoblot replicates (two independent) are shown for neuronal differentiation markers (RET, DLG2) in SK-N-BE(2) cells. GAPDH was employed as immunoblotting loading control. Quantification was performed with ImageJ and analyzed by *Student t*-test (unpaired, two-tailed). Exact *p*-values are indicated.

**Supplementary Figure 4. (A)** Additional immunoblot replicates (two independent) are shown for CLB-GE cells treated with AMXT-1501 (0 or 8 µM) and lorlatinib (0 or 16 nM) as indicated for 24 h. Lysates were immunoblotted with anti-ALK, pY1278-ALK, pAKT(S473), AKT, pERK (T202/Y204), ERK, GAPDH, PARP (asterisk (*) marked cleaved PARP), γH2A.X and α-tubulin. GAPDH and α-tubulin were employed as immunoblotting loading controls. Quantification was performed with ImageJ and analyzed by *Student t*-test (unpaired, two-tailed). Exact *p*-values are indicated. **(B-C)** The effect of AMXT-1501 and RA combination treatment on neurite length and branch points normalized to cell-body cluster area in SK-N-BE(2) (B) and SH-SY5Y cells (C). **(D)** Additional immunoblot replicates (two independent) are shown for neuronal differentiation markers (RET and DLG2) in SK-N-BE(2) and SH-SY5Y cells treated with AMXT-1501 (0 or 8 µM) and RA (0 or 5 µM) for 48 h or 24 h (n=3). β-actin was employed as immunoblotting loading control. Quantification was performed with ImageJ and analyzed by *Student t*-test (unpaired, two-tailed). Exact *p*-values are indicated.

**Supplementary Figure 5. (A-C)** Running score plots of Gene oncology GSEA analysis of RNA-seq dataset analyses. (A) NB tumors from either *Alk-F1178S;Th-MYCN* mice or *Rosa26_Alkal2;Th-MYCN* mice compared to *Th-MYCN* mice (55). (B) NB1 cells treated with either ALKAL2 or lorlatinib for 24 h (55). (C) CLB-BAR cells treated with lorlatinib for 24 h compared to DMSO treated control (39). **(D)** Additional immunoblot replicates (two independent) are shown for NB1 cells treated with ALKAL2 (1 µg/ml) and lorlatinib (0 or 30 nM) for 24 h. Lysates were immunoblotted for anti-pALK (Y1278), ALK, SLC7A5, SLC7A11, GAPDH, SLC1A5 and SLC38A1. GAPDH was employed as immunoblotting loading control. Quantification was performed with ImageJ and analyzed by *Student t*-test (unpaired, two-tailed). Exact *p*-values are indicated. **(E)** Additional immunoblot replicates (two independent) are shown for CLB-BAR cells treated with lorlatinib (0, 30 nM) for 0, 6 and 24 h as indicated. Lysates were immunoblotted for anti-pALK (Y1278), SLC1A5, SLC7A11, SLC38A1, SLC7A5 and GAPDH as indicated. GAPDH was employed as immunoblotting loading control. Quantification was performed with ImageJ and analyzed by *Student t*-test (unpaired, two-tailed). Exact *p*-values are indicated.

**Supplementary Table 1**

The worksheets list individual data points, analysis methods, and exact *P*-values for group comparisons.
