# Supplementary material for: Anaplastic Lymphoma Kinase signaling stabilizes SLC3A2 expression via MARCH11 to promote neuroblastoma cell growth
Source: Cell Death Differ. 2024 Jun 10;31(7):910–23. doi: 10.1038/s41418-024-01319-0 (PMC11239919; doi:10.1038/s41418-024-01319-0)

Supp Figure 1

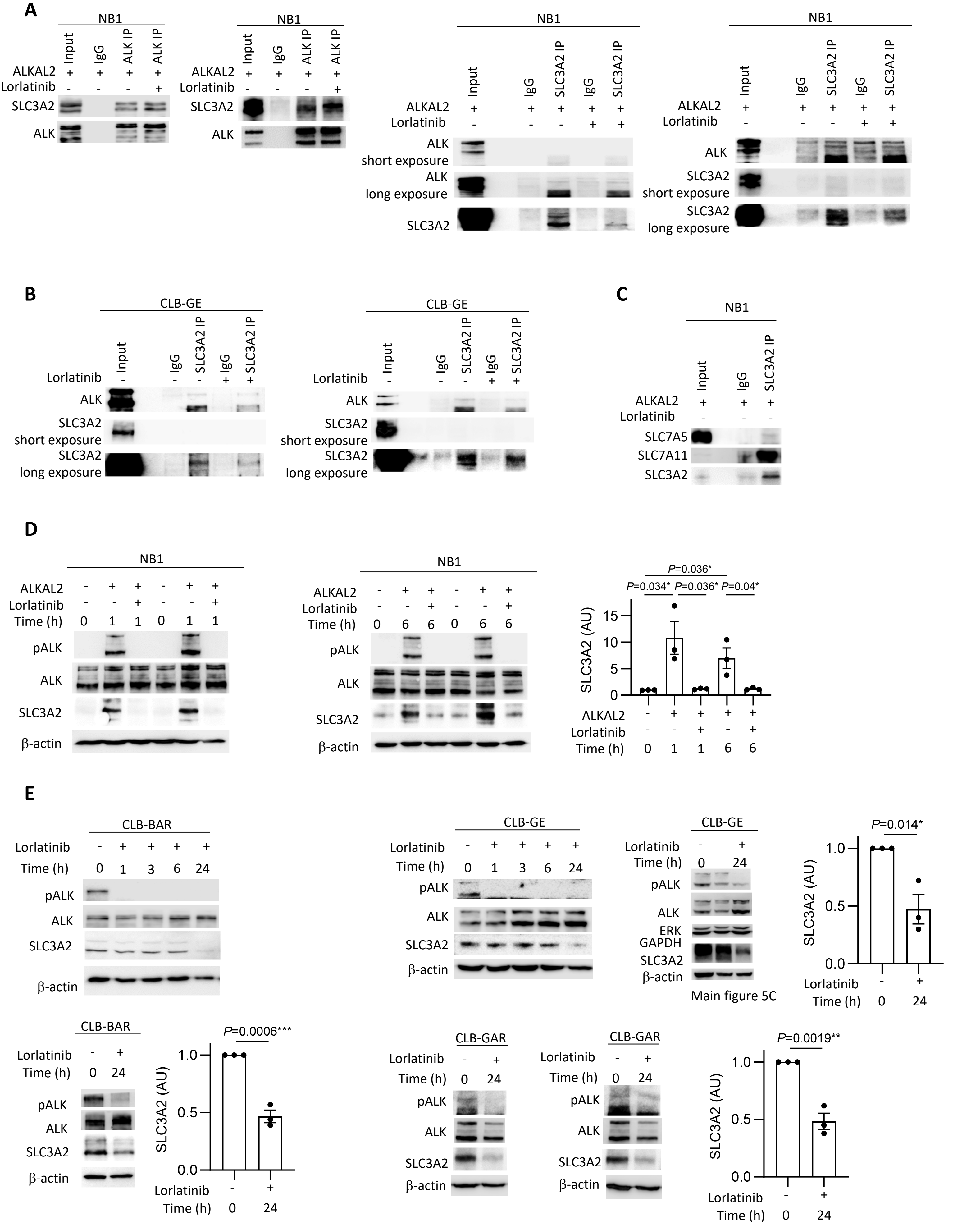

Supp Figure 1, continued…

F

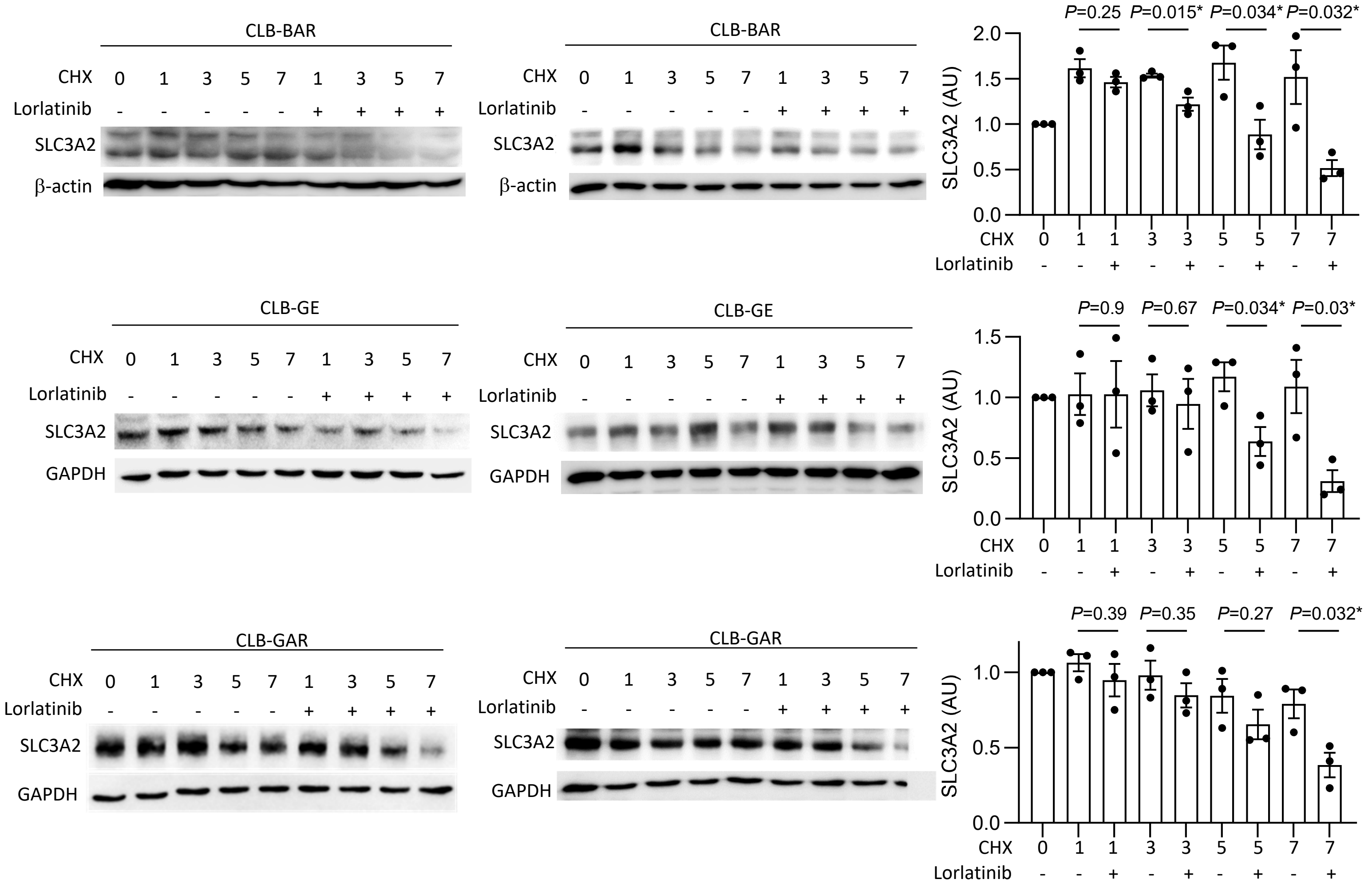

G

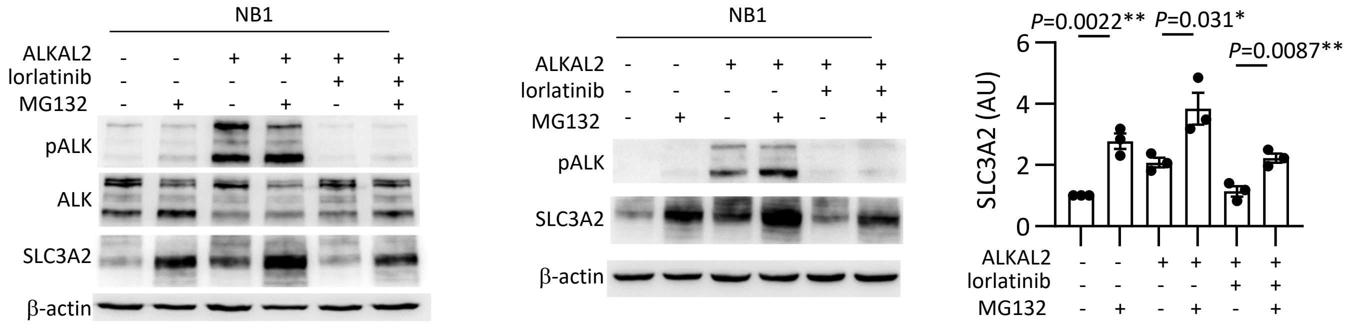

H

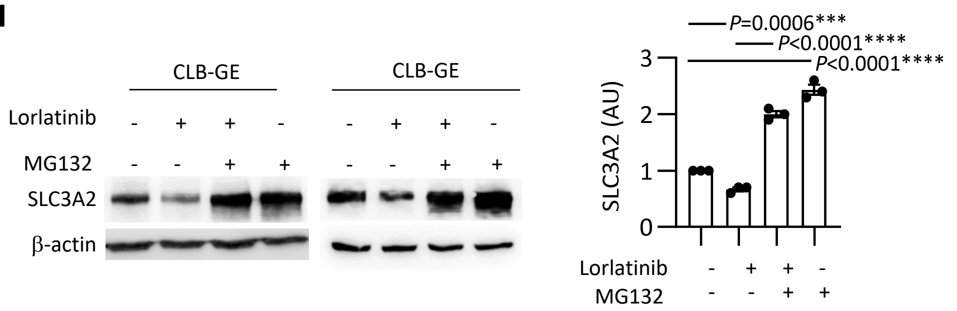

Supp Figure 2

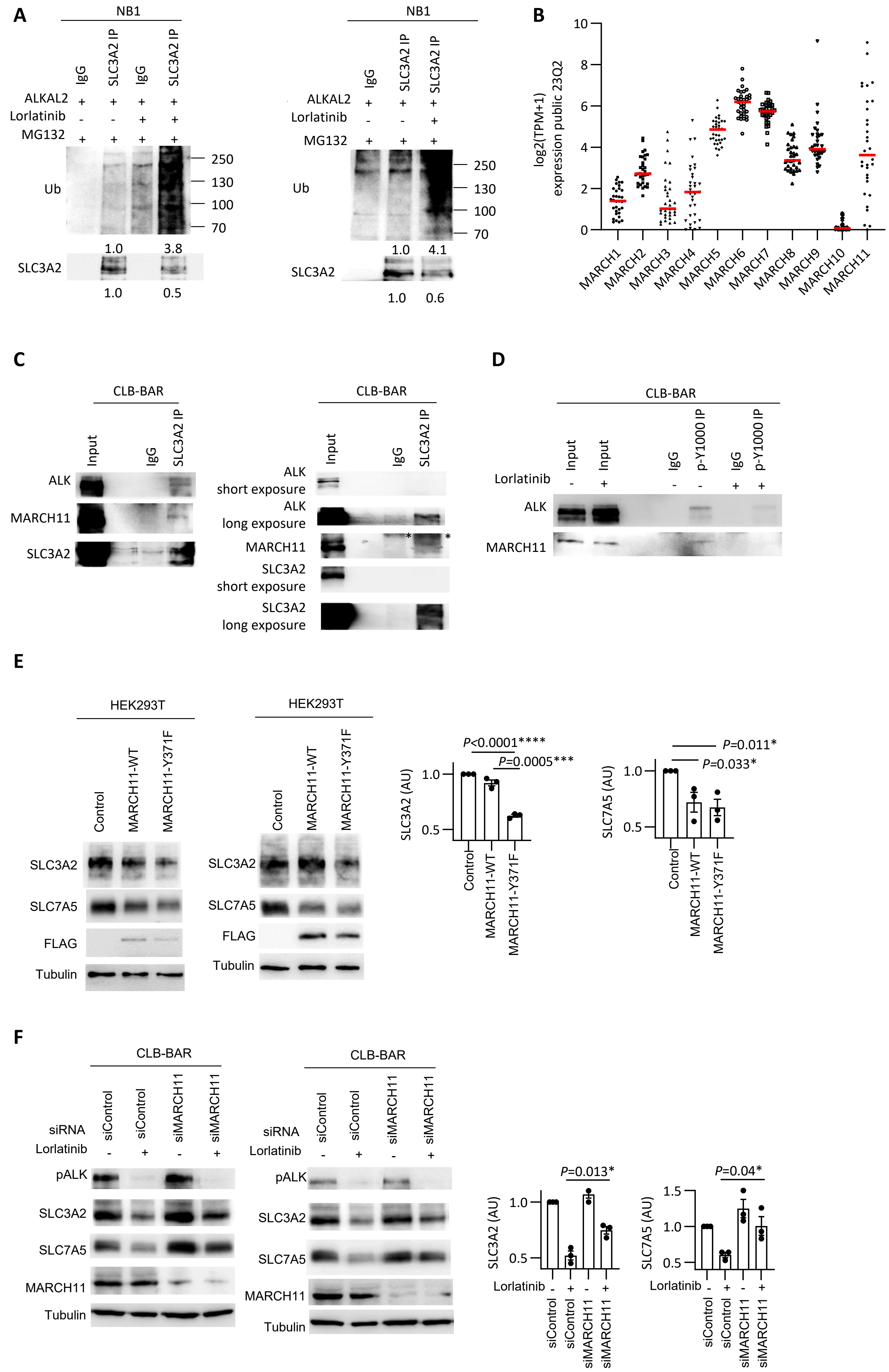

Supp Figure 3

A

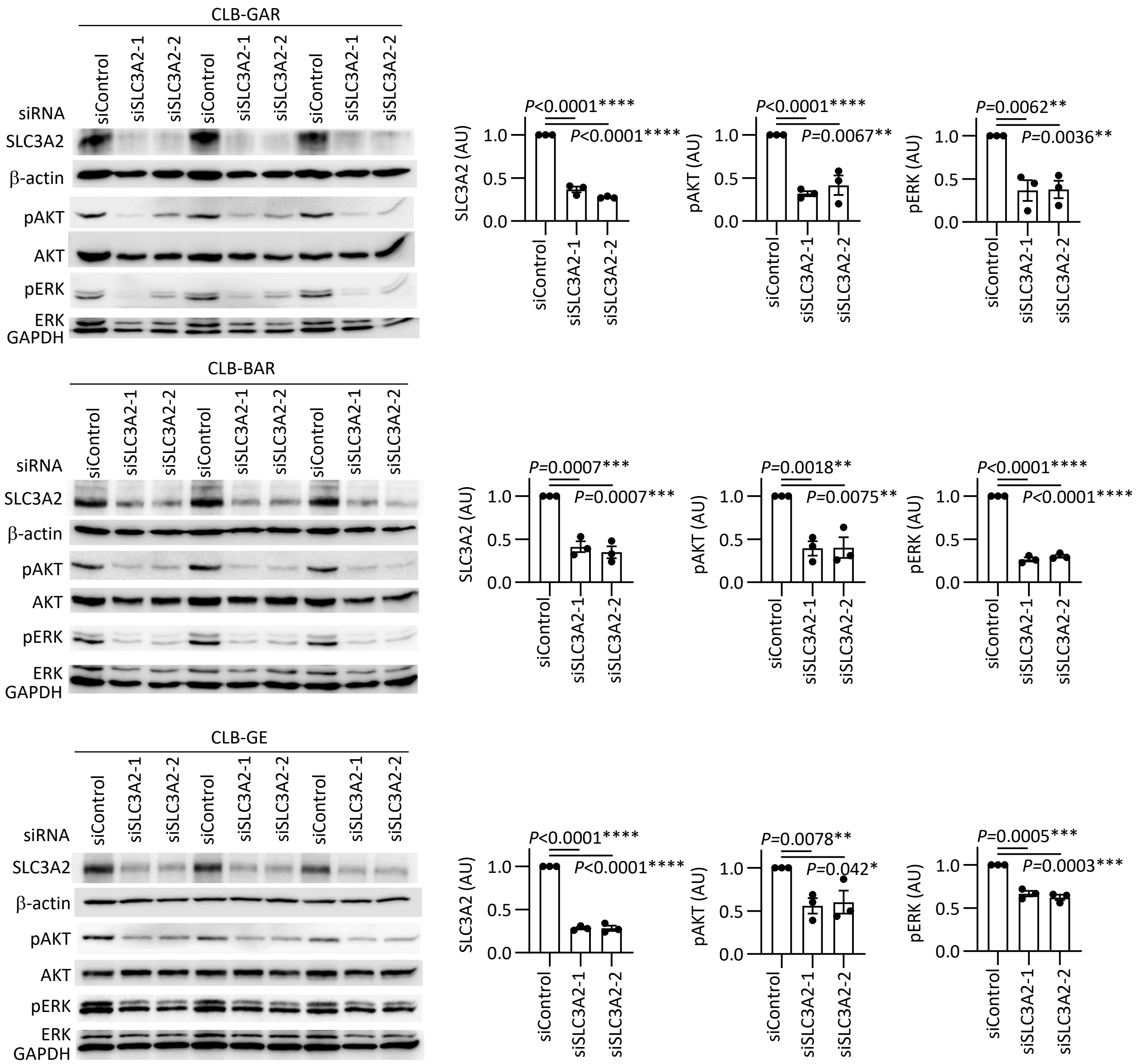

B

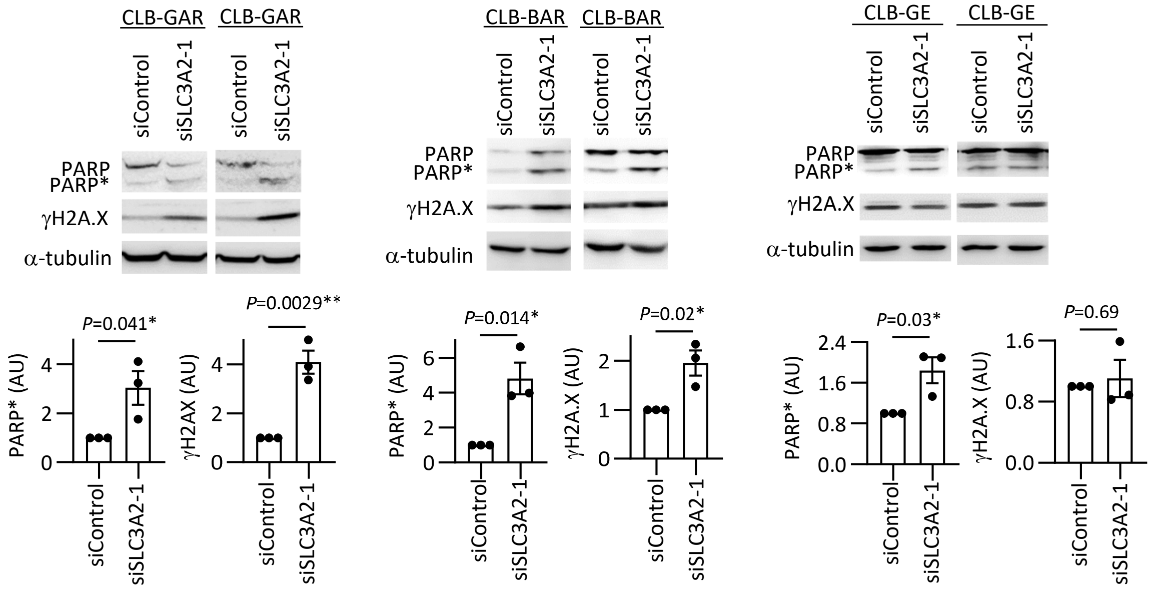

Supp Figure 3, continued…

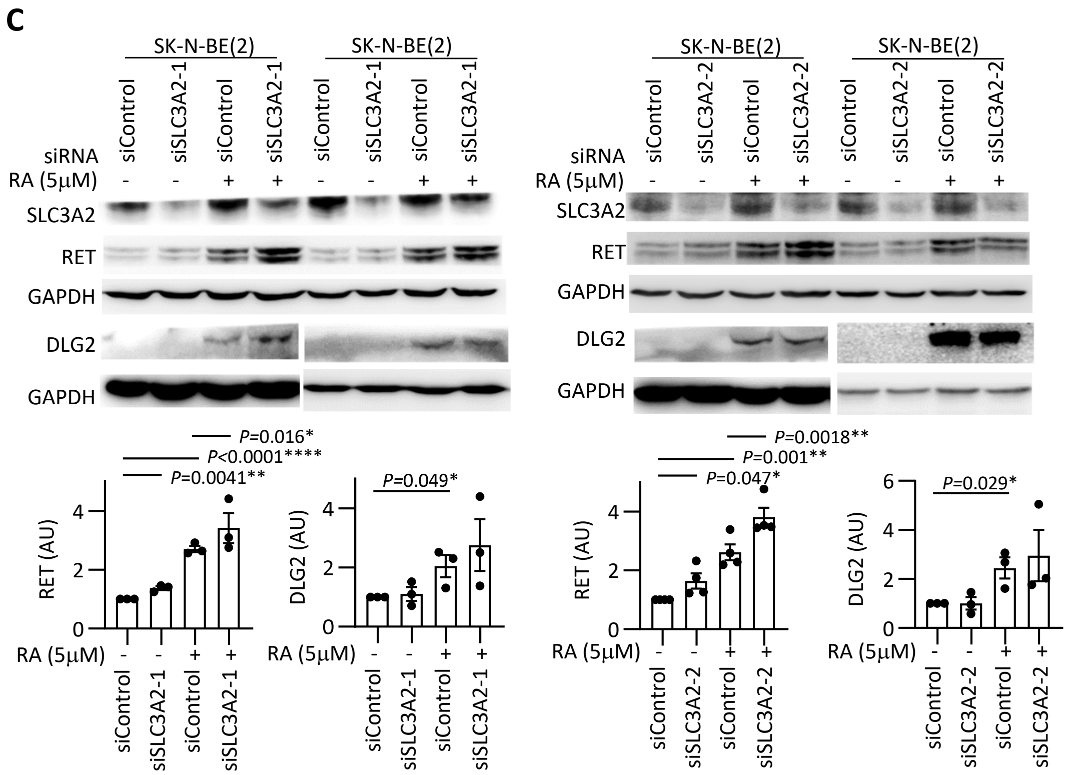

Supp Figure 4

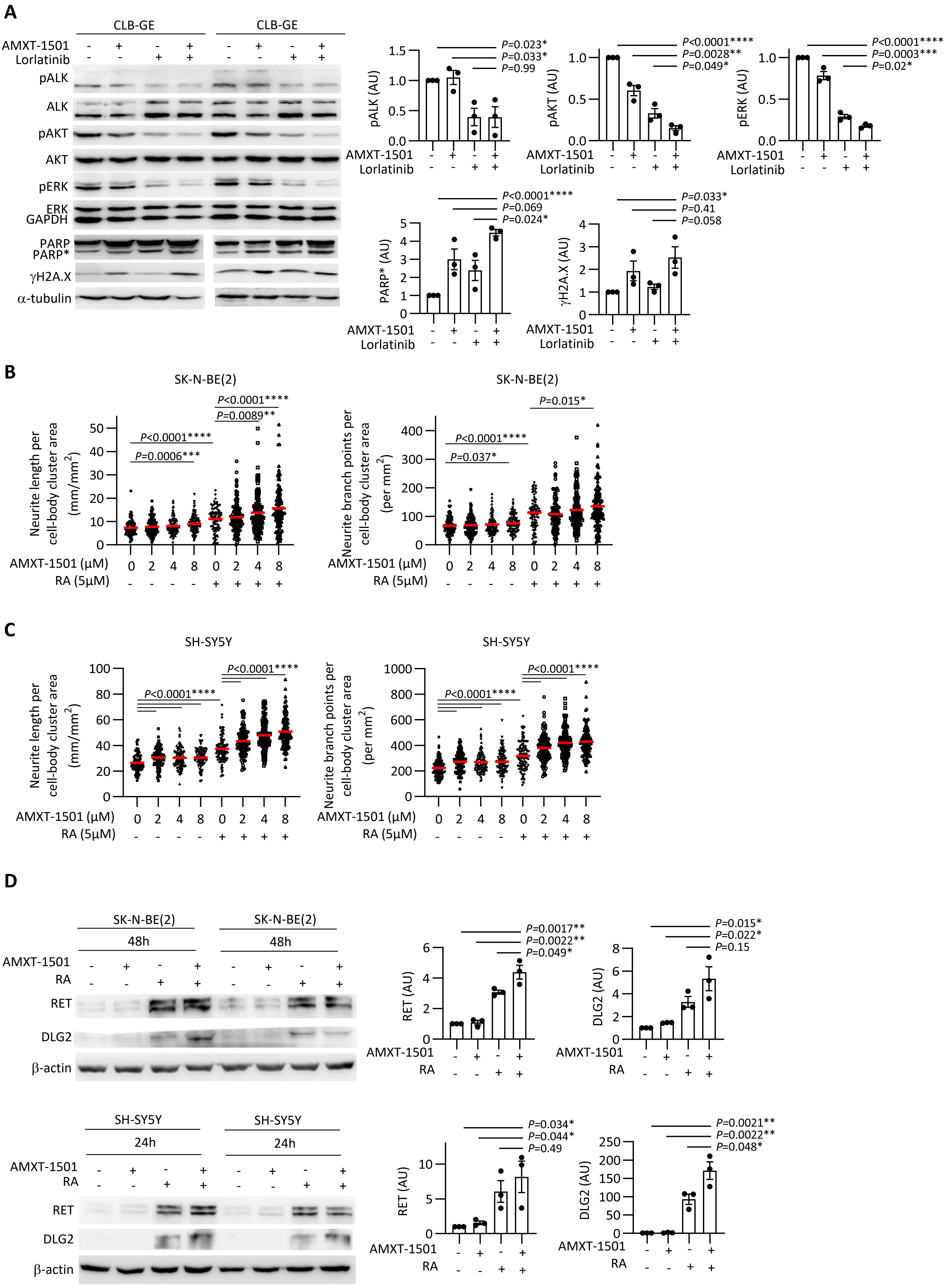

Supp Figure 5

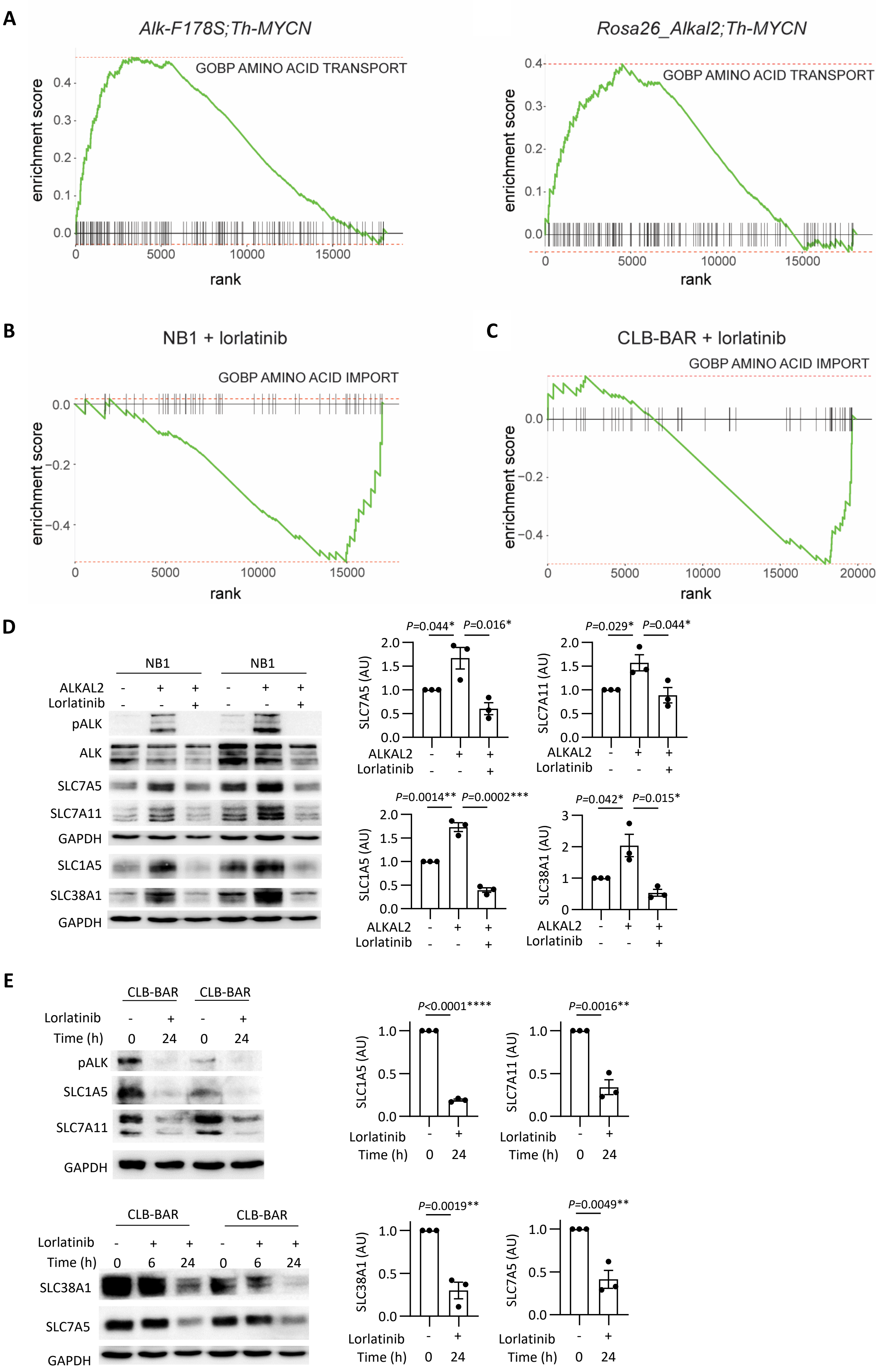

Supplement: Supplementary file 2 — Supplementary Figures [file 41418_2024_1319_MOESM2_ESM.pdf]
