# Supplementary material for: Anaplastic Lymphoma Kinase signaling stabilizes SLC3A2 expression via MARCH11 to promote neuroblastoma cell growth
Source: Cell Death Differ. 2024 Jun 10;31(7):910–23. doi: 10.1038/s41418-024-01319-0 (PMC11239919; doi:10.1038/s41418-024-01319-0)

**Uncropped blot-figure 1**

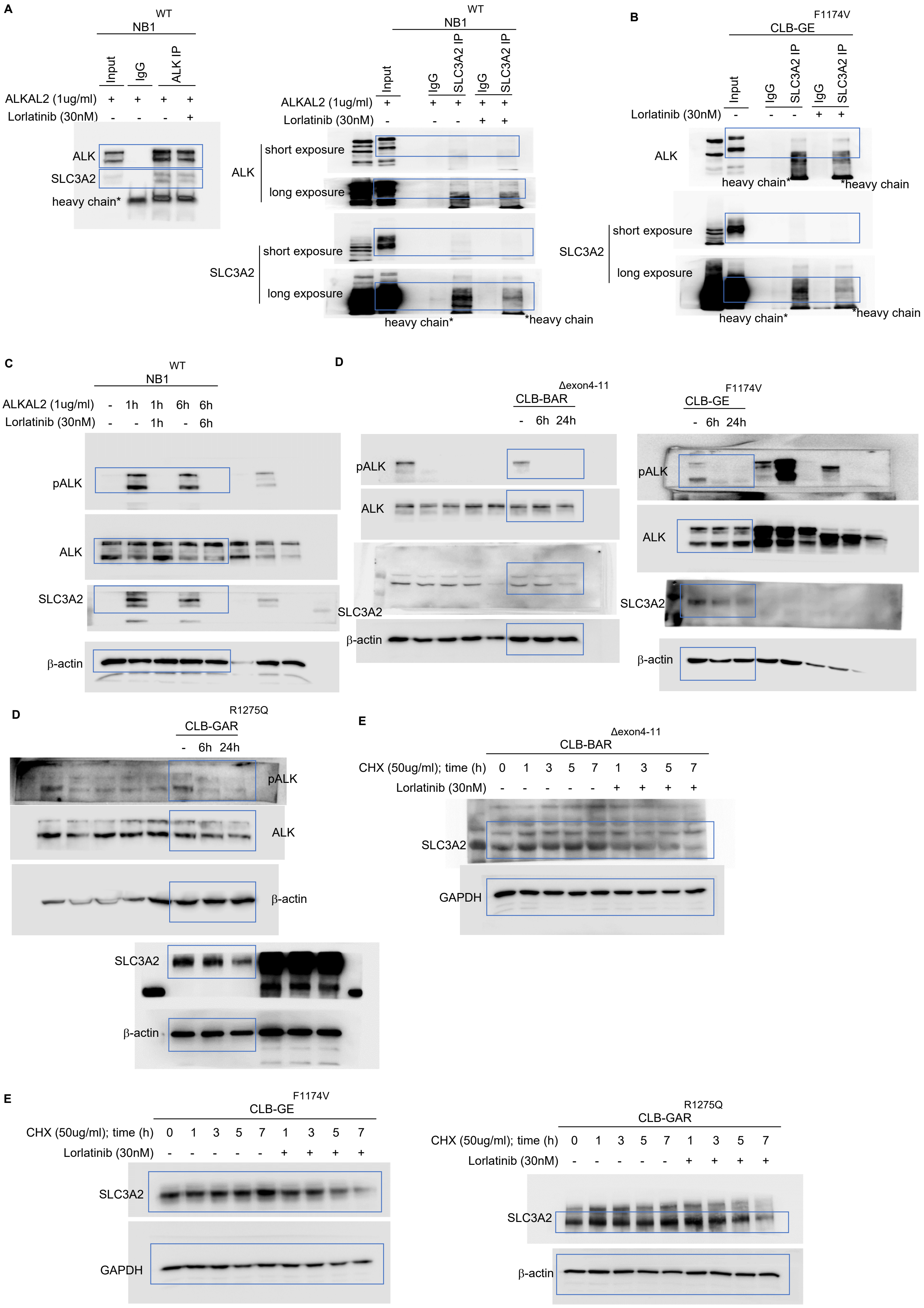

**Uncropped blot-figure 1**

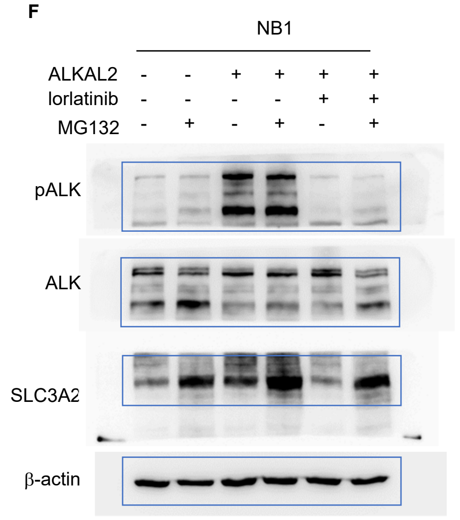


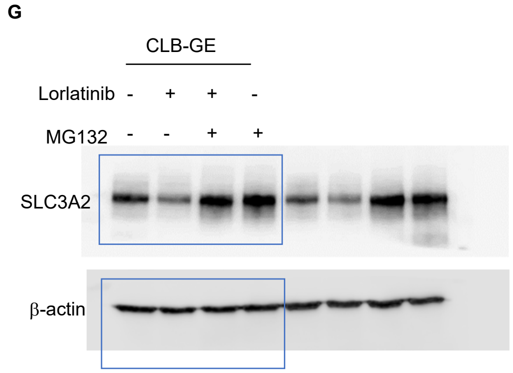

**Uncropped blot-figure 2**

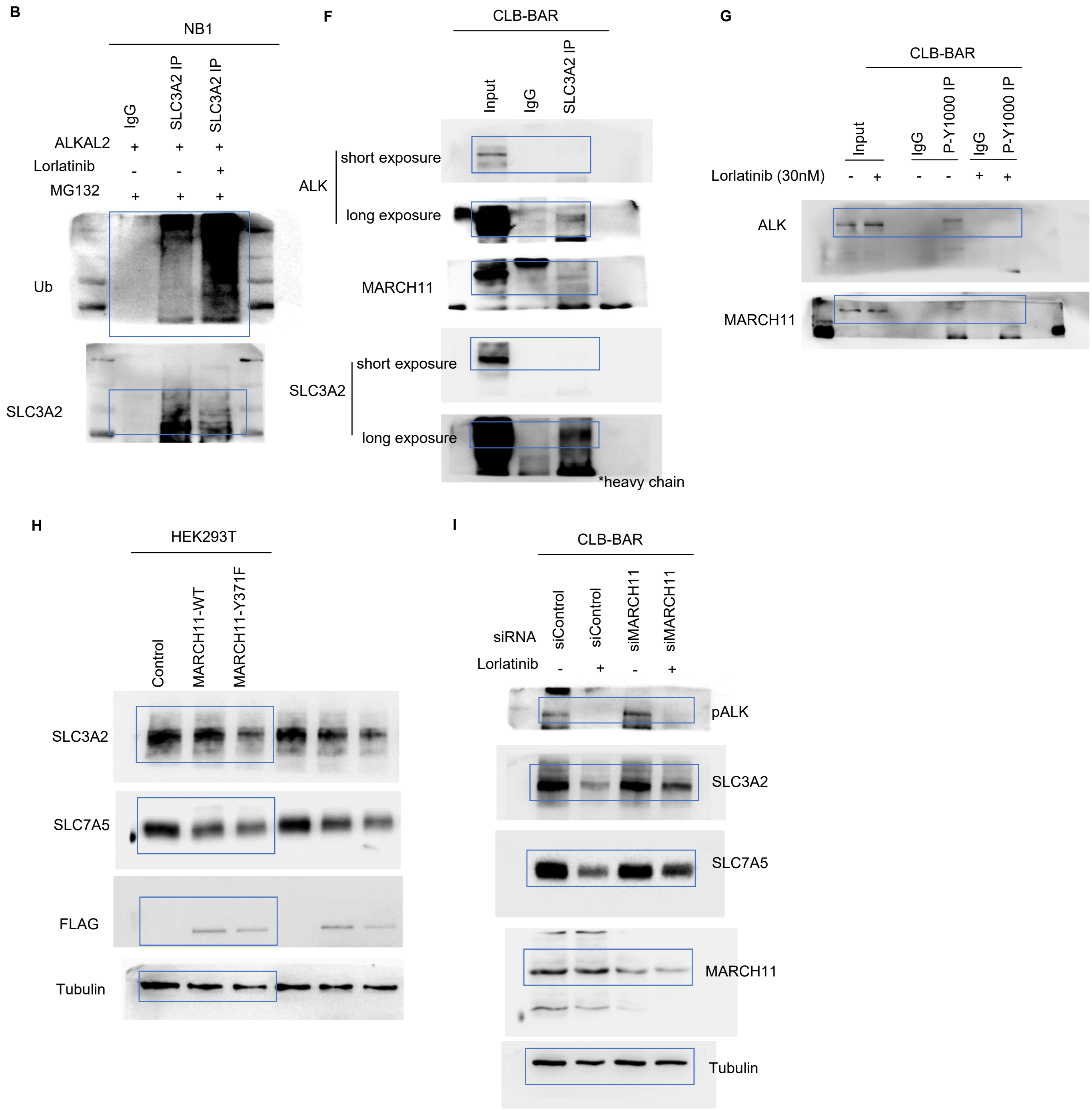

**Uncropped blot-figure 3**

**B**

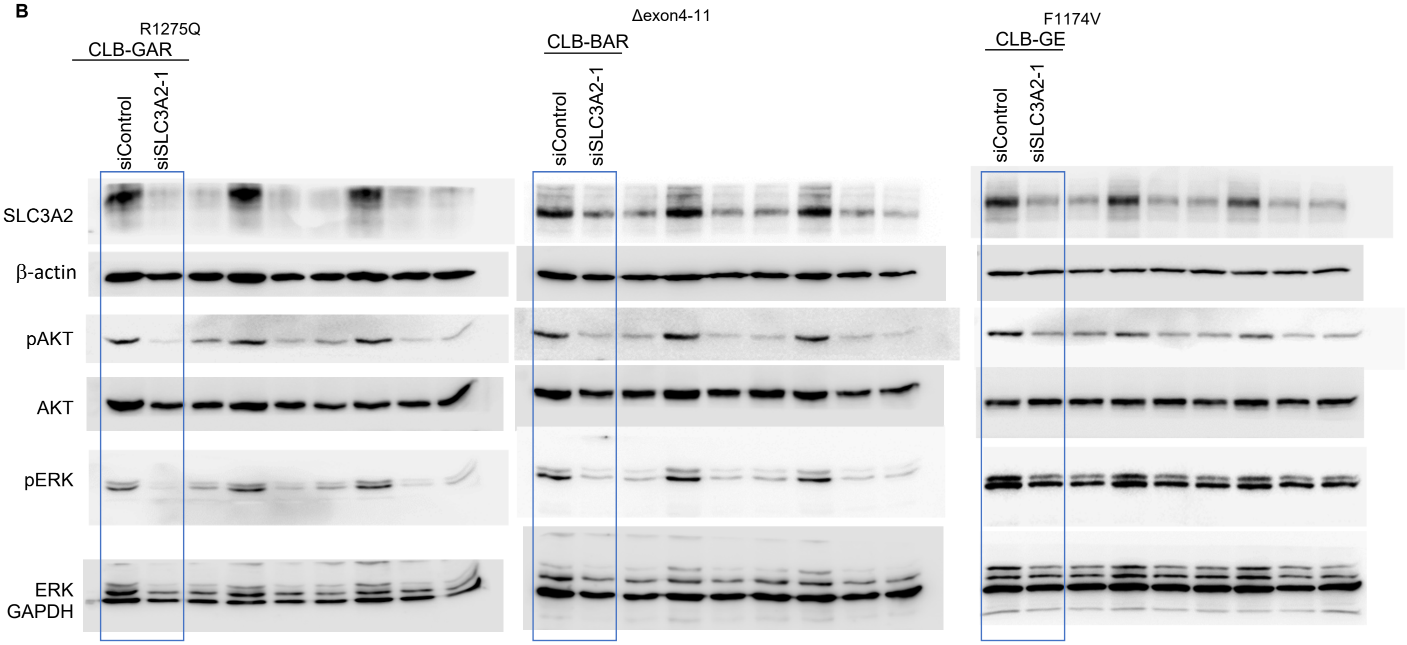


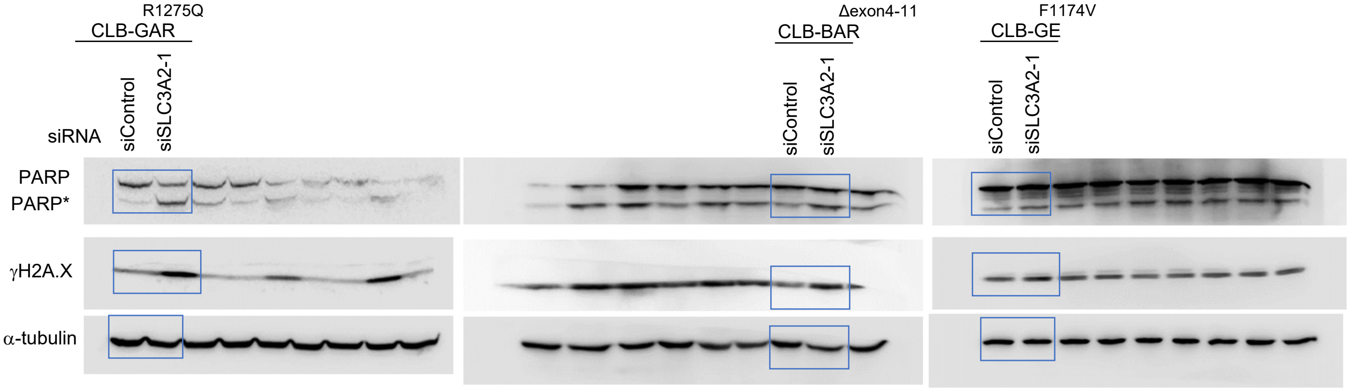


**F**

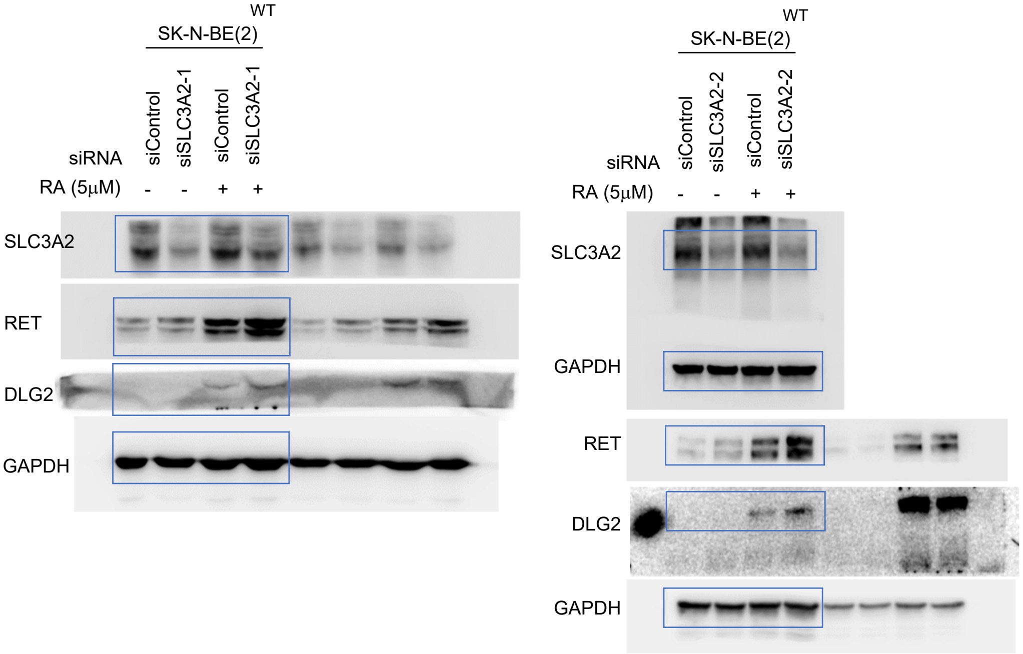

**Uncropped blot-figure 5**

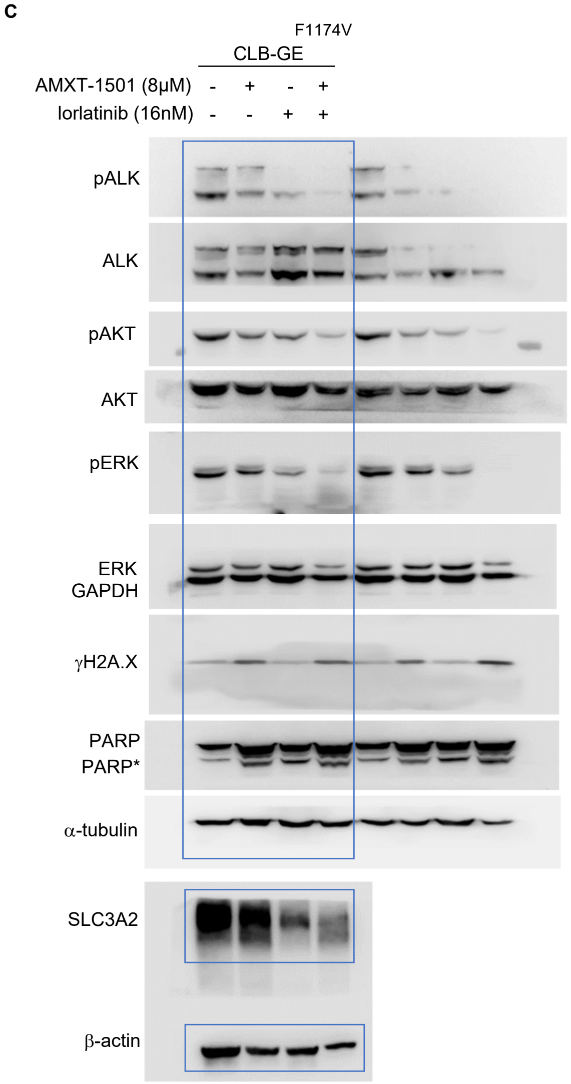


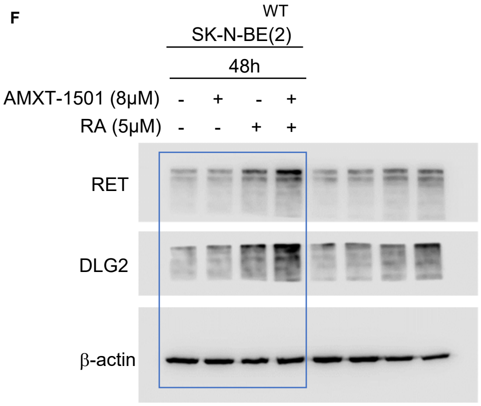


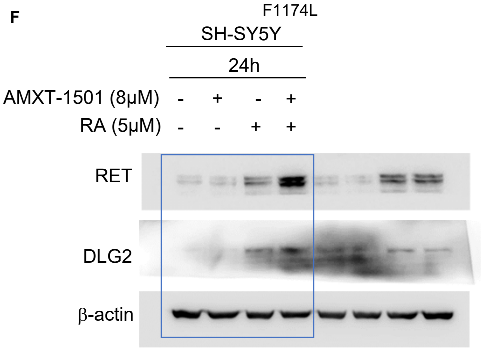

**Uncropped blot-figure 6**

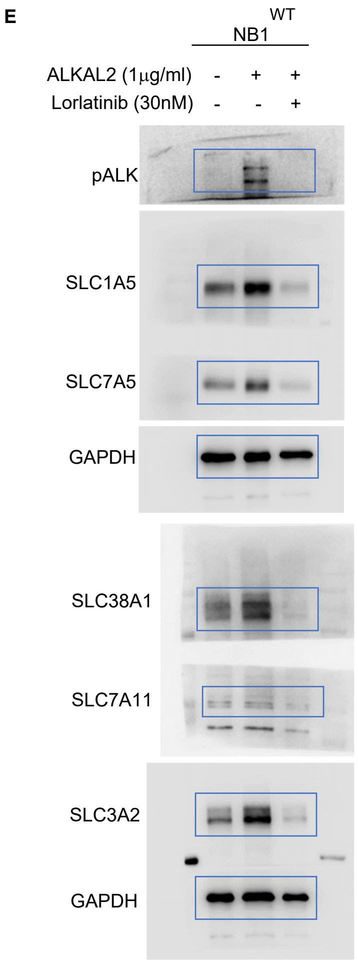


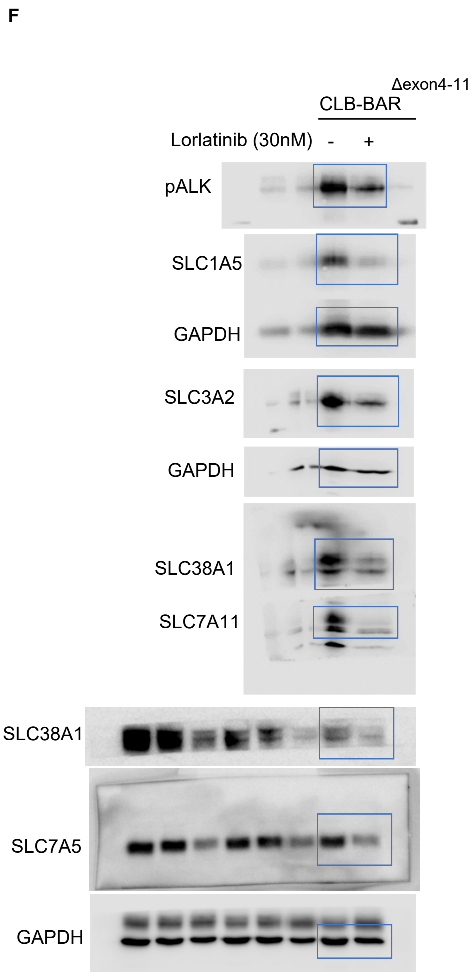

Supplement: Supplementary file 4 — Uncropped western blots [file 41418_2024_1319_MOESM4_ESM.pdf]
